# Cancer-associated fibroblast-derived WNT2 increases tumor angiogenesis in colon cancer

**DOI:** 10.1007/s10456-019-09688-8

**Published:** 2019-10-30

**Authors:** Daniela Unterleuthner, Patrick Neuhold, Katharina Schwarz, Lukas Janker, Benjamin Neuditschko, Harini Nivarthi, Ilija Crncec, Nina Kramer, Christine Unger, Markus Hengstschläger, Robert Eferl, Richard Moriggl, Wolfgang Sommergruber, Christopher Gerner, Helmut Dolznig

**Affiliations:** 1 Institute of Medical Genetics, Medical University of Vienna, Währinger Straße 10, 1090 Vienna, Austria; 2 Institute of Analytical Chemistry, University of Vienna, Währinger Straße 38, 1090 Vienna, Austria; 3 Ludwig Boltzmann Institute for Cancer Research, Währinger Straße 13a, 1090 Vienna, Austria; 4 Institute of Cancer Research, Medical University of Vienna, Borschkegasse 8, 1090 Vienna, Austria; 5 Institute of Animal Breeding and Genetics, University of Veterinary Medicine Vienna, Veterinärplatz 1, 1210 Vienna, Austria; 6 Boehringer Ingelheim RCV GmbH & Co KG, Dr. Boehringer-Gasse 5-11, 1130 Vienna, Austria; 7 Present Address: CeMM, Research Center for Molecular Medicine of the Austrian Academy of Sciences, Lazarettgasse 14, 1090 Vienna, Austria; 8 Present Address: Department for Companion Animals and Horses, University of Veterinary Medicine, Veterinärplatz 1, 1210 Vienna, Austria; 9 Present Address: Servier Pharma, Tuškanova 37, 10 000 Zagreb, Croatia; 10 Present Address: Biotechnology, University of Applied Sciences, FH Campus Wien, Helmut- Qualtinger-Gasse 2, 1030 Vienna, Austria

**Keywords:** Angiogenesis, 3D co-culture, Heterotypic cell–cell interactions, WNT2, Tumor stroma, Colorectal cancer

## Abstract

WNT2 acts as a pro-angiogenic factor in placental vascularization and increases angiogenesis in liver sinusoidal endothelial cells (ECs) and other ECs. Increased WNT2 expression is detectable in many carcinomas and participates in tumor progression. In human colorectal cancer (CRC), WNT2 is selectively elevated in cancer-associated fibroblasts (CAFs), leading to increased invasion and metastasis. However, if there is a role for WNT2 in colon cancer, angiogenesis was not addressed so far. We demonstrate that WNT2 enhances EC migration/invasion, while it induces canonical WNT signaling in a small subset of cells. Knockdown of WNT2 in CAFs significantly reduced angiogenesis in a physiologically relevant assay, which allows precise assessment of key angiogenic properties. In line with these results, expression of WNT2 in otherwise WNT2-devoid skin fibroblasts led to increased angiogenesis. In CRC xenografts, WNT2 overexpression resulted in enhanced vessel density and tumor volume. Moreover, WNT2 expression correlates with vessel markers in human CRC. Secretome profiling of CAFs by mass spectrometry and cytokine arrays revealed that proteins associated with pro-angiogenic functions are elevated by WNT2. These included extracellular matrix molecules, ANG-2, IL-6, G-CSF, and PGF. The latter three increased angiogenesis. Thus, stromal-derived WNT2 elevates angiogenesis in CRC by shifting the balance towards pro-angiogenic signals.

**Electronic supplementary material:**

The online version of this article (10.1007/s10456-019-09688-8) contains supplementary material, which is available to authorized users.

## Introduction

Colorectal cancer (CRC) is the third most common cancer accounting for about 9% of cancer-related deaths worldwide [1]. It is now widely accepted that the tumor stroma plays a pivotal role in tumor development, progression, drug resistance, and relapse of cancer [2–4]. In CRC, stromal signatures are associated with poor prognosis and predicted resistance to chemotherapy [5, 6]. The tumor microenvironment is primarily composed of fibroblasts, immune cells, blood vessels, and extracellular matrix (ECM). The ECM, which is mainly produced and organized by fibroblasts, apart from being a structural scaffold for tissue architecture, can modulate tissue homeostasis, cell movements, and viability [7, 8]. It acts as a reservoir for growth factors and cytokines which can be released upon matrix remodeling and cleavage [9, 10]. Cancer-associated fibroblasts (CAFs) are important regulators of cancer initiation and progression as they produce cytokines, proteases, and growth factors, thereby altering inflammatory responses, cell motility, and proliferation as well as ECM deposition and remodeling [11–13].

A protein frequently upregulated in gastrointestinal cancers [14–16], but also in other cancers, such as cervical [17], pancreatic [18], and lung cancer [19], is Wingless-type MMTV integration site family member 2 (WNT2). Wnt signaling is crucial for intestinal development and homeostasis, while aberrant activation of the Wnt/ß-catenin pathway is a major driver of intestinal carcinogenesis [20–23]. In comparative studies of stromal and epithelial compartments of normal colon and CRC, CAFs were found to be the main producers of stromal WNT2 [24, 25]. WNT2 expression in CAFs led to autocrine activation of canonical Wnt signaling and enhanced fibroblast motility, which in turn positively affected invasive and metastatic potential of colon cancer cells [24].

Despite its role during lung [26] and cardiac [27] development, as well as in cancer progression, WNT2 is an important factor in placenta vascularization [28]. Moreover, WNT2 is implicated to be an angiogenic growth factor promoting liver regeneration [29–31]. Per definition, angiogenesis describes the formation of new blood vessels by expansion of the surrounding vascular network, a central process in development and wound healing. In tumors, oxygen and nutrients become limited when they reach a size of a few millimeters [32]. Building the tumor’s own access to the blood system is mainly accomplished by sprouting angiogenesis [33]. The angiogenic process involves breakdown of the basal lamina and ECM, proliferation and migration of endothelial cells (ECs), sprouting and branching of new vessels, and vessel maturation. Angiogenesis is kept in balance by the presence of pro- and anti-angiogenic factors, while changes of this equilibrium can lead to turning on the angiogenic switch in tumors, a prerequisite for tumor growth and metastasis [34, 35]. Of note, tumor angiogenesis is not only mediated by tumor cells, but also by CAFs and immune cells in the tumor stroma [36–39].

In this study, we use human umbilical vein endothelial cells (HUVECs) as well-established model system for angiogenic processes in combination with a novel angiogenesis assay [40], which allows to address the impact of WNT2 expression in colon CAFs on tumor vessel development. We show that Wnt2-derived from stromal CRC-CAFs enhances angiogenesis by increasing EC migration and invasion and by altering the CAF secretome towards pro-angiogenic factors and ECM remodeling signals. Clinically, Wnt2 expression positively correlates with vessel marker expression and lower relapse-free survival.

## Material and methods

### Cell culture

Cells were cultivated in a humidified incubator with 5% CO2 under normoxic conditions at 37 °C. Primary human umbilical vein endothelial cells (HUVECs, pooled, ATCC® #PCS-100-013™) were grown in endothelial growth medium (EGM™ 2 MV; #CC-3202, Lonza, Basel, Switzerland) and only early passages (< p6) were used. CAFs from colon cancer patients were established previously [21] and were propagated in EGM™ 2 MV. In this study, CAFs derived from two different patients were used. hTERT-immortalized foreskin fibroblasts (hTERT-BJ1, #4001-1, Clontech Laboratories, Palo Alto, CA, USA) and 293T cells (ATCC® #CRL-3216™) were cultured in Dulbecco’s modified Eagle’s medium (DMEM, #21969-035, Gibco™, Life technologies, Carlsbad, CA, USA) supplemented with 10% fetal bovine serum (FBS, #10500-064, Gibco™, Life technologies) and 2 mM l-Glutamine (#17-605E, Lonza).

### Ectopic expression of GFP and WNT proteins

293T cells were transduced with lentiviral particles for empty vector controls (EVC), WNT3A, WNT5A, and WNT2 (#LPP-NEG-Lv151-200, #LPP-T9336-Lv151-200, #LPP-B0116-Lv151-200, #LPP-G0265-Lv151-200, GeneCopoeia, Rockville, MD, USA) and were selected with 0.7 g/l G418 (#A1720, Merck, Darmstadt, Germany). For establishment of WNT2-overexpressing HUVECs and hTERT-immortalized BJ1 skin fibroblasts, pLKO.1-CMV-WNT2 and for HUVEC-GFP pLKO.1-CMV-turboGFP lentiviral particles were employed. Three different batches of HUVECs were used and selected with 2 µg/ml Puromycin (#P8833, Merck), BJ1 with 1 µg/ml.

### Co-culture angiogenesis assay

For angiogenesis studies, a microcarrier bead fibroblast co-culture assay was performed as described in [40]. In brief, collagen-coated Cytodex™ 3 microcarrier beads (#17-0485-01, Amersham, GE Healthcare, Buckinghamshire, UK) were covered with HUVECs as described in [41]. The following day 4 × 10^5^ fibroblasts and 20 HUVEC-covered beads were seeded into each well of collagen-coated 24-well plates in 1 ml of EGM™-2 MV:DMEM/10% FBS (ratio 1:10). The co-cultures were incubated for 14 days, if BJ1 fibroblasts were used, or 7 days with CAFs, changing the medium every other day. Cultures were fixed and stained immunohistochemically for CD31 and analyzed semi-automatically using ImageJ.

### 7TGP reporter assays

HUVECs at an early passage were transduced with lentiviral particles, which were produced using 7TGP reporter plasmids (#24305, Addgene, Cambridge, MA, USA). For the reporter assays, 5 × 10^4^ HUVEC-7TGP were co-cultured with 1.5 × 10^4^ 293T cells expressing either no WNT protein (EVC), WNT2, WNT3A, or WNT5A in a 24-well plate. Activation of canonical Wnt/β-catenin signaling was analyzed by determination of GFP^+^ cells using flow cytometry analysis. HUVEC-7TGP cells were stained with PE-conjugated anti-CD31 (#FAB3567P, R&D systems) at 1:500 prior to flow cytometry analysis.

### Cell proliferation assay

Cell cycle progression analysis was performed with the Click-iT™ Plus EdU Alexa Fluor™ 488 or Alexa Fluor™ 647 Flow Cytometry Assay Kits (#C10633 or #C10635, Invitrogen, Thermo Fisher). For single cultures, 10 × 10^5^ HUVEC-GFP and HUVEC-WNT2 were seeded into 6-well plates. To assess differences in cell cycle progression in co-cultures of HUVECs and CAFs, 6 × 10^4^ HUVECs were seeded together with 3 × 10^4^ CAF-NTC or CAF siWNT2 into 12-well plates and single cultures of CAFs and HUVECs as controls. After 24 h, cells were incubated with 10 µM of 5-ethynyl-2′-deoxyuridine (EdU) for 1 h at 37 °C. Cells were harvested and stained according to the manufacturer’s protocol using 7-Aminoactinomycin D (7-AAD, #A1310, Invitrogen™, Thermo Fisher) or 4′,6-Diamidin-2-phenylindol (DAPI, #D1306, Invitrogen™, Thermo Fisher) for DNA staining. Co-cultures were additionally stained with a PE-conjugated CD31 antibody (#FAB3567P, R & D Systems, Minneapolis, MN, USA; 1:500) before analysis.

### Transwell migration and invasion assays

2.5 × 10^4^ HUVECs in 120 µl of Endothelial Basal Medium (EBM™-2, #CC3156, Lonza) were seeded into a transwell insert (5.0 µm pore size, #3421, Corning, Kennebunk, ME, USA), and allowed to migrate towards 500 µl of complete EGM™2 MV. After 6 h of incubation, non-migrated cells were removed with a cotton swab from the upper part of the transwell and inserts were fixed with Roti®-Histofix 4% (Roth, Karlsruhe, Germany) for 10 min at room temperature. Transwell inserts were stained in 500 µl of 0.03% crystal violet solution. To assess invasive capacity of HUVECs, basement membrane extract (BME)-coated transwells (Cytoselect™ 24-well Cell Invasion Assay, #CBA-110, Cell Biolabs Inc., San Diego, CA, USA) were used following the manufacturer’s protocol. 2.5 × 10^4^ HUVECs were used per invasion insert and invasion was measured after 8 h of incubation. 5 pictures per insert were taken and analyzed.

### Immunohistochemistry staining of angiogenesis assays

For immunohistochemical (IHC) stainings, cells were fixed with Roti®-Histofix 4% (Roth, Karlsruhe, Germany), permeabilized and blocked in 1 × PBS/0.5% Tween20/1% BSA (#BE-17-512F, Lonza/#A1389.1000, Applichem, Darmstadt, Germany/Albumin Fraktion V pH 7,0; #A1391.0100, Applichem). The co-cultures were stained for CD31 (1:500, #M0823, Dako, Glostrup, Denmark and biotinylated anti-mouse, 1:1000, #BA-2000, Vector Laboratories, Burlingame, CA, USA) using streptavidin-conjugated horseradish peroxidase (#RE7104-CE, Leica Biosystems, Nussloch, Germany) and AEC + substrate chromogen (#K3461, Dako). Images were captured with an Olympus IX51 microscope (Olympus GmbH, Hamburg, Germany). IHC overview images were taken using a CL 1500 ECO stereomicroscope (Carl Zeiss, Oberkochen, Germany) and a ToupCam™ camera (UCMOS, ToupTek Photonics, Zhejiang, China).

### Correlation of WNT2 mRNA expression and vessel markers

For WNT2 and EC marker (PECAM1 [CD31], CDH5 [VE-Cadherin), KDR [VEGFR2]) correlation studies in human colon cancer samples, individual expression levels were extracted from the TCGA-COAD, READ and GSE14333, GSE39582 datasets using Xenabrowser [42], or GEO2R (https://www.ncbi.nlm.nih.gov/geo/geo2r/) respectively, and plotted using GraphPad Prism. Linear regression analysis was performed. Pearson correlation was determined

### Survival data

Survival analysis of human CRC patients was performed in the GSE14333 and GSE39582 datasets obtained from PROGgeneV2 [43] with WNT2 and PECAM1 gene expression bifurcated at median expression into high versus low. Cumulative overall survival rates were calculated by the Kaplan–Meier method. Differences were analyzed by the log-rank test.

### siRNA-mediated knockdown

siRNA-mediated knockdown of WNT2 in CAFs was conducted as described previously [44] using Lipofectamine® RNAiMAX Transfection Reagent (#13778075, Life technologies, Carlsbad, CA, USA) and SMARTpool ON-TARGETplus siRNA (WNT2: #L-003938-00-0005, Dharmacon, Lafayette, CO, USA). Cells transfected with ON-TARGETplus Non-targeting Pool (#D-001810-10-05, Dharmacon) served as controls (non-targeting control: NTC).

### RT-qPCR

RNA was isolated with the ReliaPrep™ RNA Cell Miniprep System (#Z6011, Promega, Fitchburg, WI, USA) and cDNA was synthesized using the GoScript™ Reverse Transcription Mix, OligodT (#A2790, Promega). mRNA levels were determined via quantitative real-time PCR using the GoTaq® qPCR Master Mix (#A6002, Promega) on an Applied Biosystems™ StepOnePlus™ Real-Time PCR System (Applied Biosystems, Waltham, MA, USA). Relative expression levels were assessed using the ΔΔCt method. Primers used: WNT2-fw-5′-CCAGCCTTTTGGCAGGGTC-3′; WNT2-rev-5′-GCATGTCCTGAGAGTCCATG-3′; GAPDH-fw-5′-AACAGCGACACCCACTCCTC-3′; GAPDH-rev-5′-CATACCAGGAAATGAGCTTGACAA-3′

### Cytokine and growth factor arrays

For harvesting cell culture supernatants, 3 × 10^5^ HUVECs and 2.5 × 10^5^ CAFs were seeded into 6-well plates. For co-cultures 2 × 10^5^ CAFs were seeded and the following day 6 × 10^5^ HUVECs were added. The next day cells were washed with 1 × PBS and cells were incubated for 24 h in EGM™2 MV without FBS and hydrocortisone. The cell culture supernatants were analyzed for differential expression of various cytokines using the Proteome Profiler Human XL Cytokine Array Kit (#ARY022, R&D Systems, Minneapolis, MN, USA) and a LEGENDplex™ Custom Panel Multi-Analyte Flow Assay Kit (#92919 Custom Panel Human, BioLegend, San Diego, CA, USA) according to the manufacturer’s protocols. The Proteome Profiler signals were determined by densitometric scanning, and background controls were subtracted from the signals. The sum of all signals from individual assays was used for normalization, the results were ranked by the mean strength of one condition, and heatmaps were generated using Microsoft Excel conditional formatting.

### Secretome profiling by high-resolution mass spectrometry

Proteins from serum-free supernatants of three biological replicates of CAF-NTC and CAF-siWNT2 were processed and a filter-based in-solution digest with a Trypsin/Lys C Mix (#V5071, Promega) was performed with 20 µg of each sample as described previously [45]. Peptides were analyzed on a Q-Exactive™-Orbitrap (Thermo Fisher Scientific, Waltham, MA, USA) coupled to a nano-HPLC system (Dionex Ultimate 3000). MS and MS/MS scans were performed as described [46]. For protein identification and label-free quantification (LFQ), the MaxQuant software utilizing the Andromeda search engine and the Perseus statistical analysis package were employed [47, 48]. Proteins were identified by searching the UniProt database. Principal component analysis (PCA) was performed with Perseus. Venn’s diagrams were generated using Venny 2.1 [49]. For gene ontology (GO) enrichment analysis, the DAVID functional annotation tool version 6.8 was used [50, 51]. A detailed description of this method is available in Supplementary Material and Methods (Supplementary File S1).

### Matrigel tube formation assay

Assessment of endothelial tube formation under treatment with 20 ng/ml of human recombinant IL6, G-CSF, PGF, or 100 ng/ml of ANG-2 (#200 06, #300 23, #100 06, #130 07, Peprotech, Rocky Hill, NJ, USA) was performed in a 96-well µ angiogenesis plate (#89646, ibidi GmbH, Planegg, Germany). 1 × 10^4^ HUVECs were seeded onto pre-plated 10 µl of growth factor reduced Matrigel® matrix (#356231, Corning Inc., Corning, NY, USA) in 70 µl of DMEM supplemented with 5% FCS and 10% of EGM™2 MV and the respective growth factor. After 11 h of incubation, the cells were stained with 4 µg/ml calcein AM (#17783, Merck) in Hanks’ balanced salt solution (HBSS, #14175-053, Gibco) for 30 min at 37 °C. Fluorescent images were analyzed with the free AngioTool software [52], available at https://ccrod.cancer.gov/confluence/display/ROB2/Home.

### Vessel density analysis in a xenograft tumor model

For xenograft experiments, tumors were re-evaluated from Kramer et al. [24] in compliance with the 3R rules for animal experimentation. In brief, 1 × 10^6^ HCT116 tumor cells ectopically expressing WNT2 or GFP were subcutaneously injected into severe combined immunodeficient (SCID) mice. Tumor volume was assessed and tumors were formalin fixed and embedded in paraffin. Sections were stained immunohistochemically with an Endomucin-antibody (#14-5851-82, eBioscience™, Invitrogen, Thermo Fisher) and quantitative image analysis was performed using the Definiens Tissue Studio software (Definiens, Munich, Germany) for semi-automatic histology image analysis.

### Statistical analysis

Data were collected in Microsoft Excel and further analyzed in GraphPad Prism. Mass spectrometry data were analyzed using Perseus and were graphically displayed in GraphPad Prism. Comparisons between differentially treated or genetically modified groups and control groups were calculated using a t test for independent samples (two tailed, unpaired) provided the data were normally distributed. Data not following a Gaussian distribution were compared with a non-parametric Mann–Whitney U test. P values ≤ 0.05 were considered statistically significant. Multiple comparisons were analyzed by one -way ANOVA using the Holm–Sidak's multiple comparisons test.

## Results

### WNT2 expression does not alter cell cycle progression

**Fig. 1:**
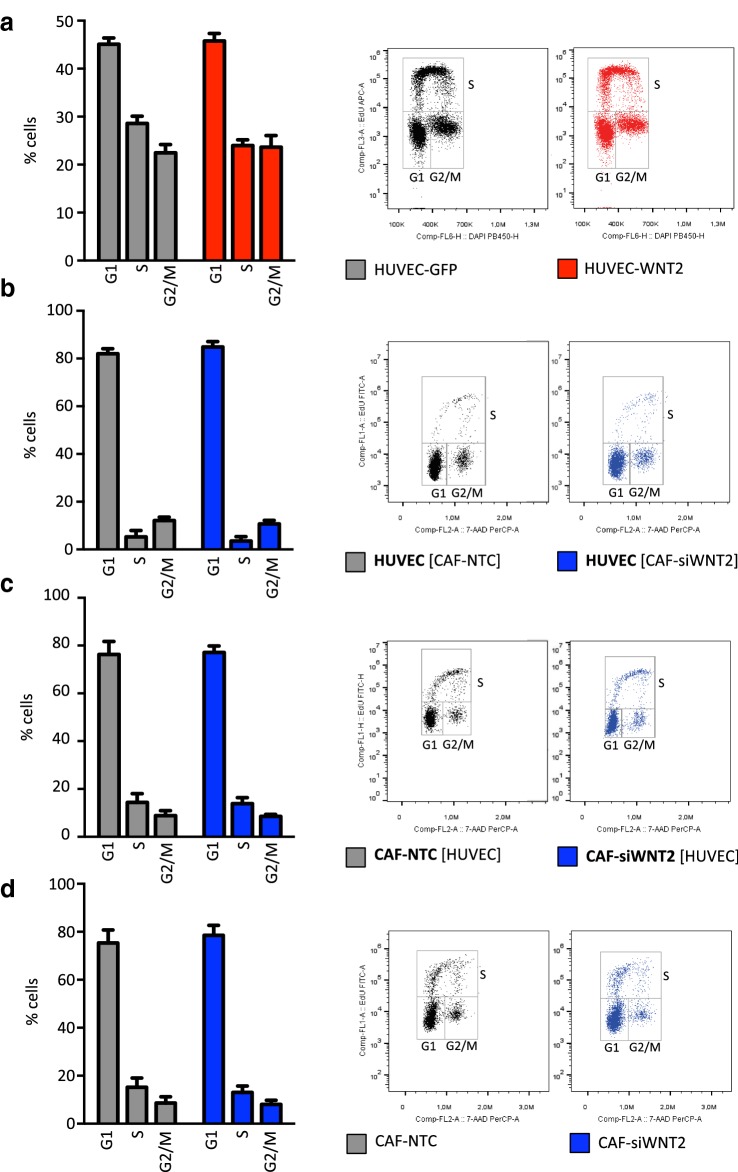
WNT2 does not change cell cycle progression in HUVECs and CAFs. Cell cycle profiles were obtained by flow cytometry analysis (right panels show one representative result) of monolayer cells by EdU incorporation (20 min pulse) and 7AAD staining. Percentages of G1, S, G2/M phase were determined (*n* = 3). Bars are mean ± SEM; data are from three biological replicates. **a** HUVECs either expressing GFP (HUVEC-GFP) or ectopically expressing WNT2 (HUVEC-WNT2) in monoculture. **b**, **c** HUVECs in co-culture with CAF either depleted of WNT2 by siRNA-mediated knockdown (CAF-siWNT2) or transfected with non-targeting control (CAF-NTC). The EC marker CD31 was used to distinguish the two cell types in the co-cultures. Cell cycle distribution of HUVEC cells (gated as CD31^+^) is shown in **b**, whereas the profiles of the CAFs (CD31^−^) are shown in **c**. Co-cultures are indicated by listing both cell types, and bold letters indicate cells analyzed; square brackets indicate the cells not being analyzed. **d** Monoculture of CAFs grown in monolayers.

As previously shown, colonic CAFs express high levels of WNT2 compared to normal colonic fibroblasts [24]. Thus, we addressed the role of WNT2 on tumor angiogenesis in colon cancer. First, the impact of WNT2 expression on proliferation and cell cycle distribution was evaluated. We expressed WNT2 in primary HUVECs and assessed the percentage of cells in G1, S, and G2/M phase by EdU incorporation and total DNA labeling. HUVEC-WNT2 displayed no changes in cell cycle distribution as compared to controls (Fig. 1a). In addition, HUVECs displayed the same cell cycle profile (Fig. 1b) when co-cultivated with colon CAFs, which endogenously express WNT2 [24] (HUVEC [CAF-NTC]), as compared to CAFs lacking WNT2 expression (HUVEC [CAF-siWNT2]). Interestingly, there was a strong reduction of proliferation in ECs upon co-culture indicating a potential differentiation effect induced by the CAFs. Furthermore, cell cycle progression was not altered in CAF-NTC and CAF-siWNT2 co-cultivated with HUVECs (Fig. 1c) or alone (Fig. 1d). The same results were obtained with other CAFs (Supplementary Figure S1).

### WNT2 induces minor canonical WNT signaling in endothelial cells and enhances HUVEC cell migration and invasion

**Fig. 2:**
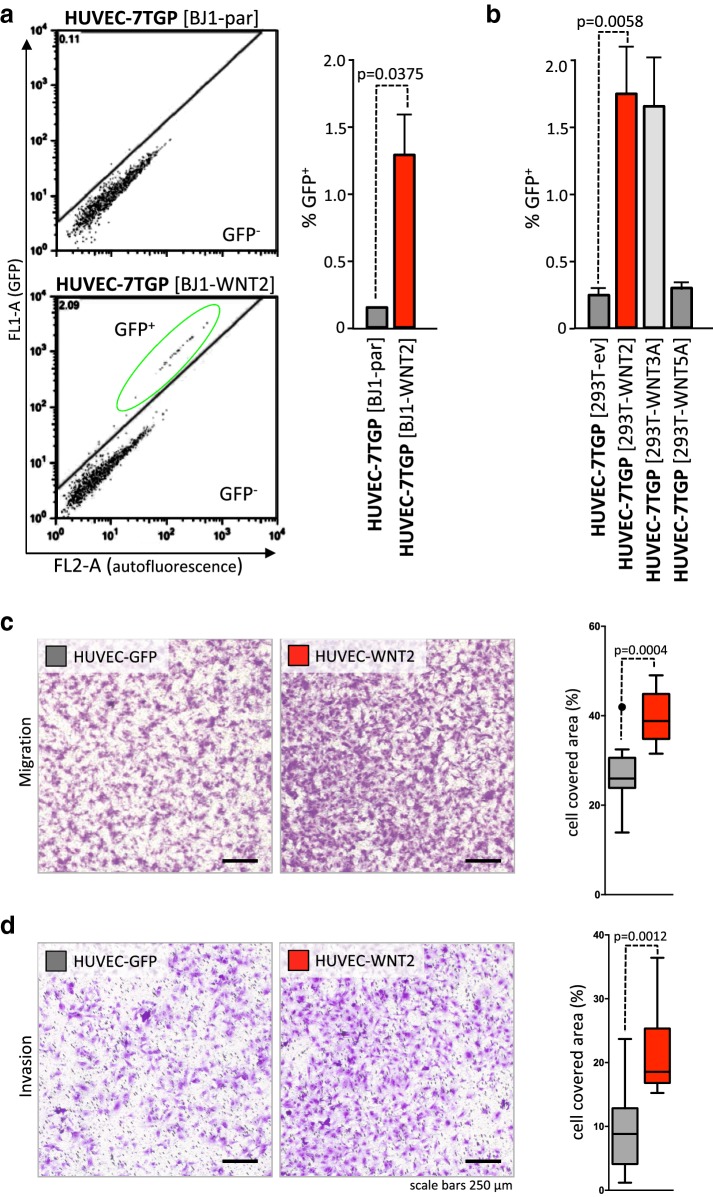
WNT2 induces canonical signaling only in a small subset of HUVEC, but significantly induces migration and invasion of HUVEC. HUVECs stably transfected with a 7TGP reporter plasmid were co-cultured with different WNT-molecule-producing cells for 72 h and WNT-induced GFP expression was monitored by flow cytometry. **a** HUVEC-7TGP co-cultured with parental BJ1 fibroblasts or BJ1 ectopically expressing WNT2. GFP^+^ gating strategy is shown (left). Mean percentage of GFP expressing HUVECs (CD31^+^) is shown right (HUVEC-7TGP [BJ1-par], *n* = 3; HUVEC-7TGP [BJ1-WNT2], *n* =  5). **b** HUVEC-7TGP co-cultured with 293T cells engineered to express WNT2, WNT3A, or WNT5A or containing empty vector control (ev). Bars indicate mean percentages of GFP^+^ cells, *n* = 4; error bars indicate SEM. P values are indicated. **c**, **d** Migration and invasion of HUVEC-WNT2 (red) from factor-free basal medium towards full EGM™-2MV in comparison to HUVEC-GFP (gray) was assessed using transwell migration inserts with 5.0 µm and 8.0 µm pore sizes, respectively (representative data of three [migration, 6 h] or two [invasion, 8 h] biological replicates performed in technical duplicates are depicted). Representative pictures of crystal violet stained migrated cells at the lower surface of the transwell membrane are shown in c (left). Quantification of migration. Membrane coverage data of HUVECs are shown in Whisker-box plots (right). Representative pictures of crystal violet stained HUVECs invading through EBM-coated inserts are depicted in **d** (left). Quantification of invasion (right). Horizontal lines in the plots indicate the median, boxes represent the interquartile range (IQR) between the 25th and 75th percentile, and whiskers extend to 1.5 times the IQR. Outliers are displayed by dots; *P* values are indicated.

Using a 7TGP luciferase reporter construct [53], the effect of WNT2 on canonical WNT-β catenin signaling in HUVECs was measured. As it has already been demonstrated that direct cell–cell contact is needed to mediate WNT2 signaling [24], co-culture experiments of cells expressing or lacking WNT2 expression were used. These cells were co-cultivated with HUVECs harboring a stable 7TGP reporter construct (HUVEC-7TGP). Skin fibroblasts overexpressing WNT2 (BJ1-WNT2) induced reporter gene activity in only about 1.4% of HUVEC, which was significantly higher than background 7TGP activation in HUVECs co-cultivated with parental BJ1s lacking WNT2 expression (Fig. 2a). Similar results were obtained when WNT2 was expressed in 293T cells (response rate in HUVEC-7TGP 1.8%). Again empty-vector-control 293T cells and 293T cells expressing non-canonical WNT5A displayed low background activation of the reporter in HUVEC-7TGP (< 0.3%). Interestingly, the *bona fide* strong activator of canonical WNT signaling, WNT3A, induced the same low response rate (1.5%) in HUVECs as WNT2 (Fig. 2b).

Next, cell migratory phenotypes of ECs were assessed using transwell assays. Ectopic WNT2 overexpression in HUVECs (HUVEC-WNT2) notably induced migration towards full EGM™2 MV medium as reflected in increased amounts of migrated cells (Fig. 2c, Supplementary Figure S2). Quantitative evaluation revealed a highly significant and up to fivefold elevation of migrated cells within six hours in HUVEC cultures derived from three different batches of pooled HUVECs (for HUVEC#1 see Fig. 2c; HUVEC#2 and #3 are shown in Supplementary Fig. 2). In all cases, HUVEC-WNT2 were compared to cells transfected with the same vector construct but expressing GFP (Fig. 2c, right). Invasive capacity was tested using the same transwell setup with basement membrane covered pores. Strikingly, there was also a significant increase of invading cells upon WNT2 overexpression in HUVECs as compared to GFP expressing cells (Fig. 2d, Supplementary Fig. 2d).

### WNT2 induces vessel growth and sprouting in a physiologically relevant angiogenesis assay

We employed a novel 3D co-culture angiogenic-sprout-formation-assay to assess the angiogenic property of stromal fibroblast-derived WNT2. This assay enables the quantification of multiple angiogenic properties of ECs being in close contact to fibroblasts comparable to the in vivo situation. We have demonstrated previously that ECs in this setup form small vessels with lumina [40].

**Fig. 3:**
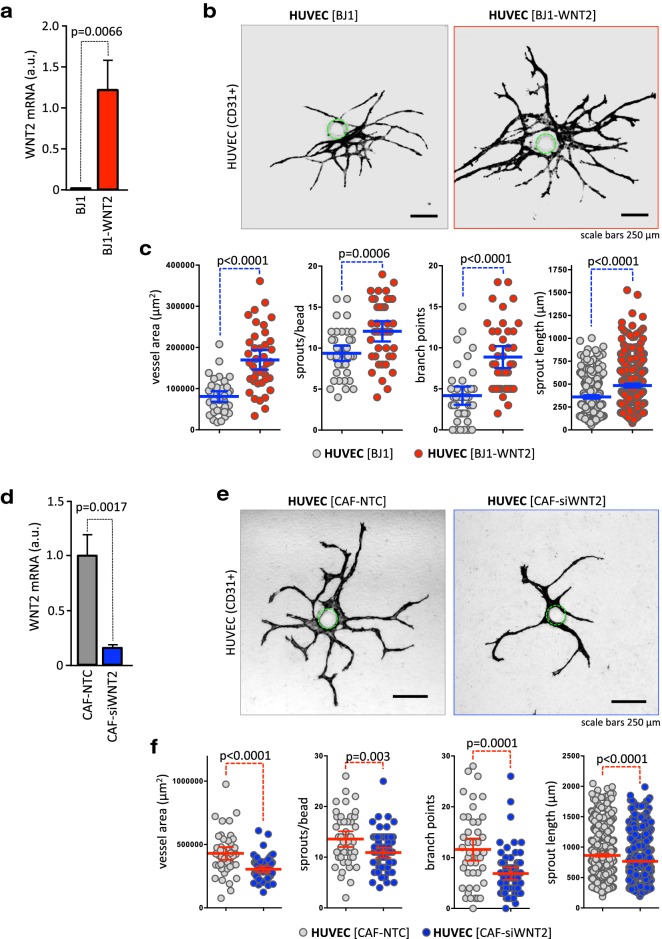
Fibroblast-derived WNT2 induces vessel growth and sprouting in a 3D angiogenesis co-culture assay. **a** Skin fibroblasts ectopically expressing WNT2 (BJ1WNT2, red) or with parental BJ1 (gray) were co-cultivated with HUVEC-coated microcarrier beads. WNT2 overexpression was evaluated by RT-qPCR. **b** After 14 days of co-culture, endothelial structures were stained with CD31 and representative images are depicted. The position of the bead is indicated by a green dotted line. **c** Image processing was used to quantify vessel areas, sprout numbers, branch points, and sprout length per bead [40]. Blue horizontal lines indicate the mean, error bars are SEM, endothelial structures derived from 40 beads were analyzed for each condition, and P values are indicated. **d** CAFs (CAF#1) endogenously expressing WNT2 (CAF-NTC, gray) or with a WNT2 knockdown (CAF-siWNT2, blue) were co-cultivated with HUVEC-coated microcarrier beads. WNT2 depletion was evaluated by RT-qPCR. **e** After 14 days of co-culture, CD31^+^ endothelial structures were evaluated and representative images are depicted. The position of the bead is indicated by a green dotted line. **f** Vessel areas, sprout numbers, branch points, and sprout length per bead were measured. Red horizontal lines indicate the mean; error bars are SEM; CAF-NTC, *n *= 45; CAF-siWNT2, *n * = 63; *P* values are given.

First, WNT2 was overexpressed in human skin fibroblasts (BJ1-WNT2), which do not express WNT2 endogenously (Fig. 3a, Kramer et al. [24]). HUVECs were co-cultured with BJ1-WNT2 and BJ1 cells as controls in the angiogenesis assay and CD31^+^ structures were evaluated after 14 days. Representative pictures of the vessel structures are shown in Fig. 3b. In co-culture with BJ1-WNT2 HUVEC displayed larger vessel structures as compared to controls. Quantitative assessment revealed highly significant enlarged vessel areas, increased sprouts/bead, and elevated branch points/bead (Fig. 3c). The length of the sprouts was also augmented.

Next, we used colonic CAFs, which endogenously express WNT2 and performed siRNA-mediated knockdown of WNT2 in these cells (Fig. 3d). Strikingly, the endothelial structures were decreased in size when WNT2 was ablated in comparison to co-cultures using CAFs transfected with non-targeting siRNA (CAF-NTC). Representative images are shown in Fig. 3e (see also Supplementary Figure S3). Quantification revealed highly significant reductions in vessel areas; sprout numbers and branch points (Fig. 3f, Supplementary Figure S4). These experiments were performed with CAFs derived from two different donors at least in triplicates using different batches of HUVECs, all displaying the same results.

Taken together, these data strongly support the hypothesis that elevated WNT2 in CAFs of colon carcinoma exerts a pro-angiogenic function as overexpression increased angiogenic properties and knockdown repressed endothelial structure formation.

### WNT2 increases angiogenesis in colorectal cancer in vivo

**Fig. 4:**
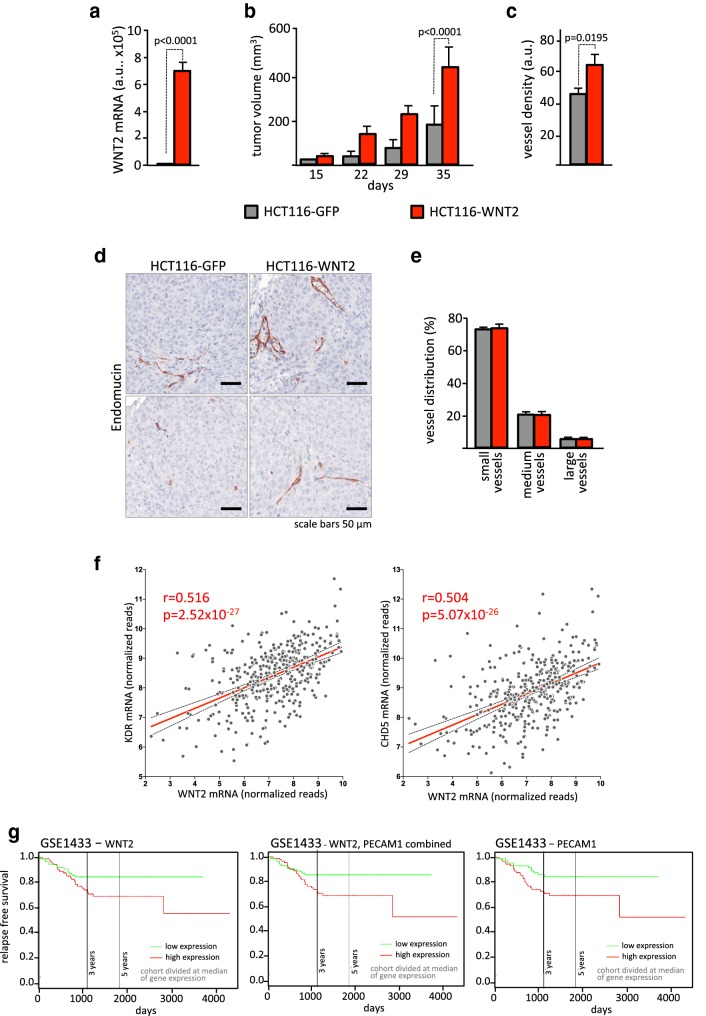
WNT2 overexpression is associated with increased angiogenesis in vivo. **a** WNT2 expression was assessed in HCT116 cells either expressing GFP (HCT116-GFP, gray) or WNT2 (HCT116-WNT2, red) by RT-qPCR analysis. **b** 1 × 10^5^ cells were subcutaneously injected into SCID mice and tumor growth was measured. **c** Vessel density was quantified by Endomucin IHC staining and image analysis using Tissue Studio. **d** Two representative images are shown. Vessels (Endomucin^+^) are stained with AEC (brown). **e** The distribution of small, medium, and large vessels within the two groups was assessed with Tissue Studio. **b**, **c***n* = 4 for HCT116-GFP and n*n* =  3 for HCT116-WNT2, and bars are means; error bars represent SEM. *P* values are indicated. **f** Correlation of WNT2 and VEGFR2 (KDR) as well as VE-cadherin (CDH5) mRNA expression in 382 CRC patients using the TCGA COADREAD RNASeq dataset. Gray dots represent individual samples; red line illustrates linear regression. Confidence interval (95%) is shown (dotted lines). Pearson’s correlations and *P* values are indicated. **g** Survival analysis of 290 CRC patients using the Sieber (GSE14333) dataset. Data were bifurcated for high and low WNT2, PECAM1, or WNT2/PECAM1 expression at the median and a Kaplan–Meier plot was generated with SurvExpress [74]. Red, high; green, low expression of WNT2, PECAM1, or WNT2/PECAM1.

In a next step, we evaluated xenograft experiments with WNT2 overexpressing HCT116 colon cancer cells that we recently published on the role of WNT2 as a driver in colorectal cancer progression [24] to investigate the in vivo relevance of our findings. Since co-injected human stromal cells rapidly disappear when co-injected with tumor cells [24, 54], WNT2 was expressed directly in the tumor cells (Fig. 4a) and tumor growth was evaluated. As reported [24], subcutaneous tumors in mice grew at increased rate when WNT2 was expressed compared to GFP controls (Fig. 4b). Interestingly, and in concordance with our in vitro data, vessel density was significantly increased in HCT116-WNT2 tumors compared to HCT116-GFP controls (Fig. 4c) as determined by Endomucin staining (Fig. 4d). Of note, the distribution of small, medium, and large vessels was not altered in the cancers indicating that WNT2 expression had a general effect on tumor angiogenesis and not on vessel maturation (Fig. 4e).

The analysis of in vivo data was extended to publicly available datasets for human CRC. WNT2 mRNA expression displayed moderate to strong positive correlations to the putative endothelial cell markers vascular endothelial (VE)-cadherin (CDH5) and vascular endothelial growth factor receptor 2 (VEGFR2, KDR) mRNA expression in the TCGA-COAD and TCGA-READ datasets (Fig. 4f) as well as to CD31 (PECAM1) mRNA expression in the Sieber (GSE14333) and Marisa (GSE39582) datasets (Supplementary Figure S5). Of note, there is no single marker uniquely specific for vessels and these markers can also be expressed on hematopoietic cells. Thus, our results indicate the possibility that elevated levels of WNT2 in human colon cancer are involved in increased tumor angiogenesis. Furthermore, we concluded that if WNT2 is critically involved in tumor angiogenesis in humans, the outcome for overall survival would be influenced by WNT2 levels similarly to PECAM1 levels, directly indicating the amount of vascularization in the tumors. Indeed, we found decreased survival rates in patients with high WNT2 expressing tumors. Intriguingly, these survival curves were very similar to the curves obtained with high versus low PECAM1 levels, displaying also very similar *P* values (Fig. 4g).

In summary, these data underscore the in vivo relevance of our conclusion drawn from the in vitro experiments, further strengthening our hypothesis that stroma-derived WNT2 is a prominent angiogenesis-promoting factor in colon cancer.

### WNT2 expression in CAFs shifts the balance towards secreted pro-angiogenic factors

**Fig. 5:**
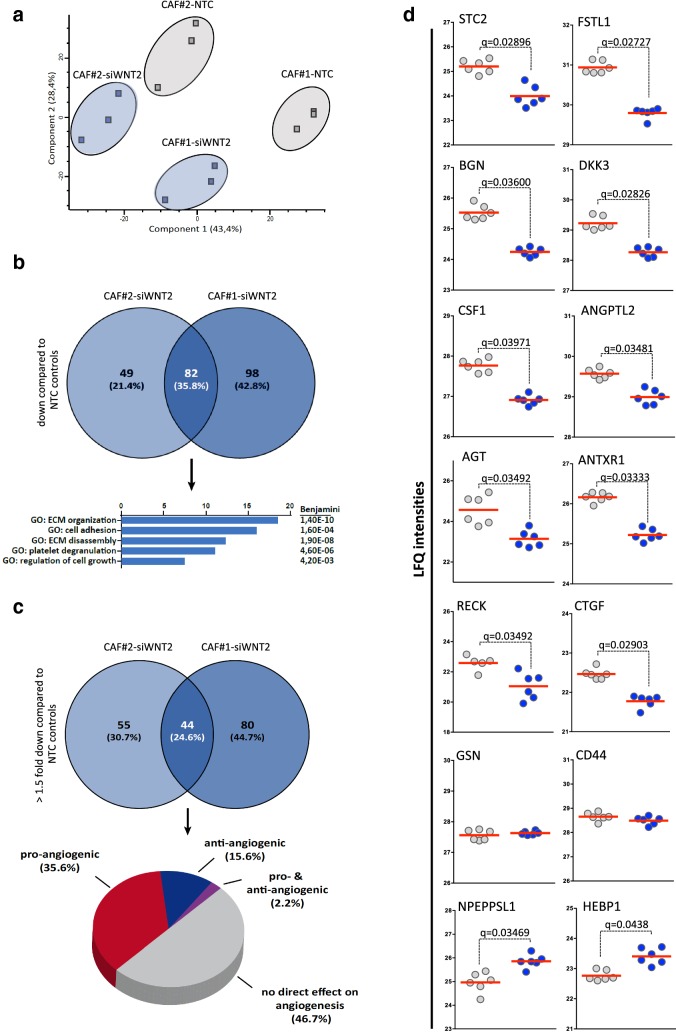
Secretome profiling of CAFs in the presence (CAF-NTC) or absence of WNT2 (CAF-siWNT2). **a** Principal component analysis of the secreted fractions of CAFs derived from two different patients treated with either non-targeting control (NTC) siRNA or with WNT2-specific siRNA. A clear distinction between the controls and the siWNT2-treated samples can be observed for both CAFs. Data were generated from three biological replicates, analyzed via LC-MS in technical duplicates. **b** Venn diagram of secreted proteins downregulated in CAFs upon WNT2 knockdown and functional annotation of the proteins with significant lower expression in both CAFs performed using the DAVID functional annotation tool. **c** Venn diagram taking only proteins significantly downregulated with a minimum fold change of 1.5 taken into account. The 44 proteins with significantly lower expression in both groups displayed in a pie chart showing the percentage of proteins reported in the literature to be directly involved in angiogenesis or not. The list of the proteins with the according references is shown in Table 1. **d** Scatter dot plots of LFQ intensities of examples for significantly down- or upregulated and not-regulated proteins in upon knockdown of WNT2 (CAF-siWNT2) compared to NTC-siRNA-treated controls (CAF-NTC) as determined by LC-MS-based secretome profiling. Data from all biological and technical replicates of CAF#1 are depicted. Red lines represent the mean. Q-values of multi-parameter corrected significance tests are indicated.

In order to get a deeper insight in the possible molecular mechanisms leading to WNT2-mediated angiogenesis, we performed qPCR-based gene expression (Supplementary Figure S6, Supplementary Tables S1). Moreover, mass spectrometry analysis was employed (Fig. 5 and Supplementary Figure S7, Supplementary Table S2) to uncover differences in secreted factors.

**Table 1:** Significantly downregulated proteins in SN of CAF-siWNT2 versus CAF-NTC from MS analysis

Gene name	Protein ID	Protein name	*P* value CAF3	*Q* value CAF3	Literature (PMID)			
Pro-angiogenic factors								
ADAM9	Q13443-2;Q13443	Disintegrin and metalloproteinase domain-containing protein 9	0.000808141	0.0427692	*29118335*			
ANGPTL2	Q9UKU9	Angiopoietin-related protein 2	0.0137859	0.0348185	*24563071*	*10473614*		
ANTXR1	Q9H6X2-3;Q9H6X2-4;Q9H6X2-6;Q9H6X2-2;Q9H6X2-5;Q9H6X2	Anthrax toxin receptor 1	0.00078728	0.0333333	*22340594*			
BGN	P21810	Biglycan	0.00122969	0.036	*24373744*	*27590684*	*22374465*	
CHI3L1	P36222	Chitinase-3-like protein 1	0.011577	0.0335	*22056877*	*24222276*		
CLU	P10909-4;P10909;P10909-5;P10909-2;P10909-3	Clusterin; Clusterin beta chain; Clusterin alpha chain	0.00850796	0.0301538	*23616046*			
CSF1	P09603;P09603-2;P09603-3	Macrophage colony-stimulating factor 1	0.00115979	0.0397143	*24892425*	*19398755*		
CTGF	P29279-2;P29279	Connective-tissue growth factor	0.00208425	0.029037	*25341039*	*28108312*		
CTHRC1	Q96CG8;Q96CG8-3;Q96CG8-2	Collagen triple helix repeat-containing protein 1	0.00201167	0.0316735	*29344195*	*27686285*		
DKK3	Q9UBP4	Dickkopf-related protein 3	0.00471913	0.0282632	*18033687*	*28352232*	*26093488*	
FSTL1	Q12841;Q12841-2	Follistatin-related protein 1	0.000566386	0.0272727	*18718903*			
GAS6	Q14393-2;Q14393;Q14393-3;Q14393-5;Q14393-4	Growth arrest-specific protein 6	0.0106919	0.0339091	*24409287*	*28627676*		
LAMB2	P55268	Laminin subunit beta-2	0.00116008	0.03104	*23571221*			
MFGE8	Q08431;Q08431-3;Q08431-2	Lactadherin;Lactadherin short form;Medin	0.00526468	0.0321633	*24838098*			
RARRES2	Q99969	Retinoic acid receptor responder protein 2	0.00154514	0.0337049	*20237162*			
STC2	O76061	Stanniocalcin-2	0.010831	0.02896	*23664860*			
Anti-angiogenic factors								
ADAMTS2	O95450-2;O95450	A disintegrin and metalloproteinase with thrombospondin motifs 2	0.00273232	0.038625	*20574651*			
AGT	P01019	Angiotensinogen;Angiotensin 1-9	0.029336	0.0364771	*19318581*	*11847188*		
IGFBP6	P24592	Insulin-like growth factor-binding protein 6	0.00694845	0.0302535	*21618524*			
SPON2	Q9BUD6	Spondin-2	0.0158907	0.0348687	*28991232*			
TIMP1	P01033	Metalloproteinase inhibitor 1	0.00033682	0.0483478	*12704667*			
Pro- and anti-angiogenic factors								
RECK	O95980	Reversion-inducing cysteine-rich protein with Kazal motifs	0.0429898	0.0349259	*20407016*	*28803732*	*24931164*	*11747814*
No direct effect on angiogenesis								
AGRN	O00468-6;O00468-7;O00468-3;O00468-5;O00468-4;O00468;O00468-2	Agrin	0.0163424	0.0329934				
C1QTNF5	Q9BXJ0	Complement C1q tumor necrosis factor-related protein 5	0.0441136	0.0442137				
C4B	P0C0L5	Complement C4-B	0.00779713	0.0358809				
CCBE1	Q6UXH8;Q6UXH8-3	Collagen and calcium-binding EGF domain-containing protein 1	0.00948764	0.0293913				
CFD	P00746	Complement factor D	0.00152939	0.0363529				
CLSTN1	O94985	Calsyntenin-1;Soluble Alc-alpha; CTF1-alpha	0.0149868	0.0381851				
CST3	P01034	Cystatin-C	0.00066365	0.0412				
EFEMP2	O95967	EGF-containing fibulin-like extracellular matrix protein 2	9.20E-05	0.024				
FAM198B	Q6UWH4;Q6UWH4-2;Q6UWH4-3	Protein FAM198B	0.00162255	0.0298333				
FBN2	P35556	Fibrillin-2	0.0115779	0.028918				
GALNT2	Q10471	Polypeptide N-acetylgalactosaminyltransferase 2; soluble form	0.0123606	0.038241				
GM2A	P17900	Ganglioside GM2 activator;Ganglioside GM2 activator isoform short	0.0092158	0.031978				
GPX3	P22352	Glutathione peroxidase 3	0.00523958	0.0292525				
LTBP2	Q14767	Latent-transforming growth factor beta-binding protein 2	0.00124036	0.04448				
NUCB1	Q02818	Nucleobindin-1	0.000352503	0.0309767				
OAF	Q86UD1	Out at first protein homolog	0.00295179	0.0367143				
PLOD3	O60568	Procollagen-lysine,2-oxoglutarate 5-dioxygenase 3	0.000695964	0.0423158				
PLTP	P55058;P55058-3;P55058-4;P55058-2	Phospholipid transfer protein	0.0235319	0.0341993				
PODN	Q7Z5L7;Q7Z5L7-2;Q7Z5L7-3;Q7Z5L7-4	Podocan	0.00803048	0.0297554				
PTGDS	P41222	Prostaglandin-H2 D-isomerase	0.0115976	0.0317143				
QSOX1	O00391;O00391-2	Sulfhydryl oxidase 1	0.0358089	0.0444505				
SVEP1	Q4LDE5	Sushi, von Willebrand factor type A, EGF and pentraxin domain-containing protein 1	0.00436406	0.0284516				

Label free quantification (LFQ) values were compared for significant diffferences using two-sided t-tests (*P*-values).* Q*-values are false discovery rate (FDR) adjusted* p*-value for multi-parameter testing. In both cases a value < 0.05 was considered as significant

Using high-resolution mass spectrometry analysis, a global analysis of secreted proteins in CAF-NTC and CAF-siWNT2 was performed using serum-free supernatants of three biological replicates of two different primary CAF cultures. The measurements were performed in technical duplicates and the results indicated a clear separation in a principal component analysis (Fig. 5a). Upon siRNA-mediated silencing of WNT2, 180 proteins were found to be significantly downregulated (*P* value < 0.05, *Q* value < 0.05) in CAF#1 and 131 proteins in CAF#2, compared to their corresponding controls. Of note, 82 proteins of these were commonly downregulated in both CAF cultures upon knockdown of WNT2 (Fig. 5b). Functional annotation revealed that most of these proteins are linked to ECM organization and disassembly, cell adhesion, platelet degranulation, and regulation of cell growth, which is in line with our previous findings or data from the literature on effects of WNT2. When applying a more stringent criterion (FC ≥ 1.5), 44 genes showed significant lower abundance rates in the supernatants of both CAFs depleted for WNT2 (Fig. 5c). These proteins were examined for previous reports on their function in angiogenesis. In concordance to the results from our in vitro angiogenesis data, 35.6% of these proteins are connected to a reported pro-angiogenic phenotype, whereas only 15.6% were associated with an anti-angiogenic function. About half of the 44 genes were not related to an angiogenic function in the literature so far (Fig. 5c). All 44 proteins and the supporting literature are listed in Table 1. The label-free quantification (LFQ) intensities of the most downregulated proteins in CAF#2 are depicted in Fig. 5d (for CAF#1 see Supplementary Figure S7). To demonstrate that proteins are not downregulated uniformly when WNT2 is depleted in CAFs, the LFQ intensities of the secreted proteins heme-binding protein 1 (HEBP1) and puromycin-sensitive aminopeptidase-like protein (NPEPPSL1) are shown as representatives for proteins that were increased upon WNT2 knockdown and Gelsolin (GSN) and CD44 as representative examples for unchanged proteins (Fig. 5d, for CAF#1 see Supplementary Figure S7).

In summary, the data from our LC-MS experiments indicate that WNT2 expression in CAFs is tipping the balance towards a more pro-angiogenic phenotype also including alterations in ECM remodeling, which is supporting the phenotype we observed in our in vitro and in vivo experiments.

### Cytokine profiling identifies IL-6, G-CSF, and PGF as WNT2-regulated genes supporting angiogenesis

**Fig. 6:**
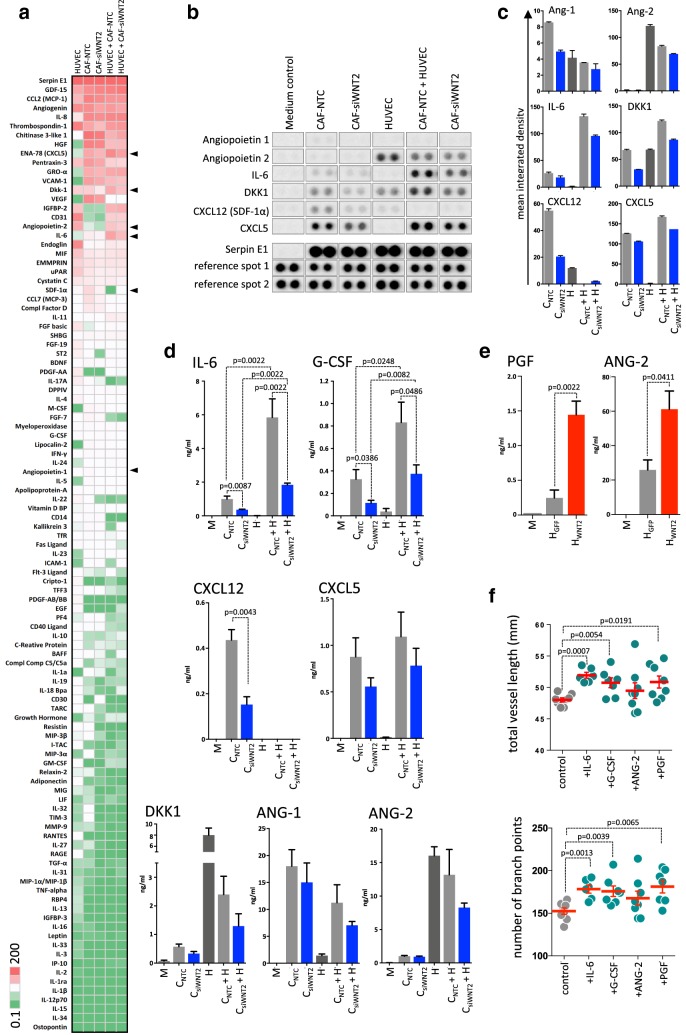
Cytokine profiling identifies IL-6, G-CSF, and PGF as WNT2-regulated genes, which support angiogenesis. **a** Semi-quantitative membrane-based cytokine array analysis (Proteome Cytokine XL Profiler) of HUVEC- and CAF-secreted factors in monocultures of HUVEC, CAF-NTC, and CAF-siWNT2 as well as in HUVEC-CAF co-cultures. Arbitrary mean expression values after densitometric quantification were ranked on average expression of all conditions from high to low and visualized as heatmap. High levels of cytokine signals are indicated in red, intermediate in white, and low or absent molecules are displayed in green. Arrowheads indicate robust differences between CAF-NTC and CAF-siWNT2 in mono- or co-cultures. **b** Actual signals of the six identified cytokines/growth factors in comparison to unchanged Serpin E1 expression and the positive controls (reference spots). **c** Mean integrated density blot of the two signals per cytokine shown in **b**. Error bars indicate range. **d**, **e** Quantitative determination of cytokines/growth factors from CAF-NTC (C_NTC_), CAF-siWNT2 (C_siWNT2_), HUVEC (H) mono- and co-cultures as well as HUVEC-WNT2 (H_WNT2_) and HUVEC-GFP (H_GFP_) cells released within 24 h into serum-free medium by a multiplex flow cytometry bead array. Mean values are shown; error bars represent SEM; *P* values are indicated for statistically significant changes. **f** Matrigel tube formation assay of HUVECs cultivated in control medium (gray) or in the presence of the indicated recombinant human factors (green; IL-6, G-CSF, PGF: 20 ng/ml; ANG-2: 100 ng/ml) for 11 h. Vessel length and the number of branch points were detected with the AngioTool software. Red horizontal lines designate the mean, and error bars are SEM; *P* values are indicated for significant changes.

As high-throughput MS analysis has reportedly some limitations in detecting low abundant and low molecular weight proteins (reviewed in [55, 56]), we extended our analysis to cytokines and growth factors being released from CAFs with or without WNT2 knockdown using antibody arrays. First, the cytokine profile of HUVECs, CAF-NTC, and CAF-siWNT2 cells in monocultures were determined and compared to co-cultures of HUVECs with control and WNT2-depleted CAFs. This analysis provides a semi-quantitative overview of the levels of 140 secreted molecules in the different cell types (Fig. 6a, Supplementary Tables S2a, b) and proteins, which displayed differences in expression (Fig. 6b, c) upon WNT2 deletion. Moreover, qPCR-based mRNA expression profiling of 352 genes, including WNT signaling molecules and targets as well as ECM genes and angiogenic growth factors (RT^2^ profiler, see Supplementary Figure S6, Supplementary Table S1), revealed a significant downregulation of granulocyte colony-stimulating factor (G-CSF, gene symbol CSF3) and placental growth factor (PGF) in CAF-siWNT2 as compared to CAF-NTC.

Flow cytometry-based bead ELISA validated the results obtained by the antibody array and the qPCR results on a quantitative level (Fig. 6d). The minor downregulation of angiopoietin 1 (ANG-1) and CXCL5 in CAF-siWNT2 as well as ANG-2 in co-cultures of HUVECs and CAF-siWNT2 was verified but did not reach significance. In support of the array data, the ELISA measurements revealed a significant downregulation of stromal-derived factor 1 (SDF-1; CXCL12) in CAFs devoid of WNT2 as compared to their controls; surprisingly, CXCL12 levels however dropped to undetectable levels when CAFs were in contact with HUVECs, which was independent of the WNT2 status in the fibroblasts. This indicates a strong repression of CXCL12 expression in CAFs by HUVECs for unknown reasons. IL-6 and G-CSF were expressed in CAFs and displayed highly significant downregulation in CAF-siWNT2. Moreover, these molecules were robustly increased in co-cultures with ECs and still displayed the WNT2-specific differences. In addition, WNT2 expression in HUVECs induced PGF and ANG-2 secretion (Fig. 6e). Thus, four WNT2-induced secreted molecules displaying significant regulation were detected: IL-6, G-CSF, ANG-2, and PGF. The addition of recombinant human IL-6, G-CSF, and PGF significantly increased tube formation in matrigel angiogenesis assays as revealed by increased vessel length and branching (Fig. 6f).

Taken together these results in addition to the mass spectrometry-based secretome analysis strongly underscore that WNT2 derived from colon CAFs supports tumor angiogenesis by increasing the secretion of pro-angiogenic factors in the microenvironment.

## Discussion

We recently identified WNT2 to be specifically upregulated in stromal fibroblasts of CRC, being an autocrine factor to promote CAF migration, ECM remodeling, and invasion, thereby, as a secondary effect, influencing tumor cell invasion and dissemination [24]. This was supported by independent reports on WNT2 function in esophageal [16] and gastric cancer [57]. In comparison to other Wnt molecules, the knowledge on WNT2 functions, apart from its role in development, is rather scarce. There is evidence that WNT2 is involved in angiogenesis, e.g., during liver regeneration and in placental vascularization [28, 31]. However, there is scarce evidence about WNT2 function in tumor angiogenesis.

In our experimental approach to study the effects of WNT2 on colon cancer angiogenesis, we only use direct cell–cell contact to assure proper induction of signaling, as WNT signaling in most cases is dependent on the direct contact of WNT-producing and responding cells [58, 59]. Moreover, we avoid using recombinant WNT2 since we have previously shown that commercially available rhWNT2 was not able to induce canonical WNT signaling in reporter cells [24]. In contrast, WNT2 led to signaling in responder cells only when in direct contact with WNT2-producing cells, while WNT2-conditioned medium had no effect [24]. This is in contrast to earlier findings that WNT2-conditioned medium can induce phenotypic changes in ECs [30]. This discrepancy could be explained by the fact that different producer cells (L-cells vs. CHO-K1) as well as growth medium were used and/or human WNT2 versus mouse WNT2 cDNA was employed to ectopically express WNT2 in these cells.

We based our study on HUVECs since these cells are the most comprehensively investigated model system for the examination of the regulation of ECs and their response to different stimuli. Thorough analysis of cell proliferation using precise determination of G1, S, and G2/M phase distribution by EdU incorporation revealed that WNT2 had no effect on cell cycle progression, neither in HUVECs nor in CAFs cultivated alone or in co-cultures. As CAFs are concerned, this is in line with our previous data [24]. On the contrary, an earlier report described that proliferation of HUVECs as well as lung and aortic ECs was induced by WNT2 [29], whereas in bovine aortic ECs no effect of WNT2 on proliferation was reported [60]. Again, as discussed above, this difference could be explained by different WNT2 species used (mouse vs. human) and/or different WNT2-producing cells employed. In addition, the use of conditioned media as source for specific proteins is prone to autocrine effects of the expressed protein on the producer cell line. Thus, this would lead to undefined effects in the responder cell line that might not be caused by the expressed recombinant protein directly. Therefore, it might be possible that WNT2 is able to induce autocrine signaling in the producer cells—leading to an altered secretome, which in turn could influence the proliferation rate of the responder cells. However, we show that alterations of the CAF secretome by depleting endogenous WNT2 expression in CAFs by siRNA do not change HUVEC proliferation. We further circumvent secondary effects by ectopic expression of WNT2 directly in HUVECs.

Interestingly, HUVECs displayed reduced proliferation when in contact to CAFs; however, this was independent of WNT2. Reduction in HUVEC cell proliferation might either be due to competition for mitogens and growth factors in the co-cultures or is attributed to an induction of differentiation and cessation of proliferation in the ECs mediated by the fibroblasts as demonstrated earlier [61]. The effects were highly reproducible as we analyzed different batches of HUVECs and CAFs from different donors leading to the same results.

WNT2 induced canonical Wnt signaling in only a small subset of HUVECs as demonstrated by a TCF/LEF reporter gene assay. This was assessed in two independent co-culture assays employing either fibroblasts (BJ1) or 293T cells as WNT2 producers. First, the low response rate was not due to ineffective WNT production since proper controls (RKO-7TGP) showed up to 55% response using the same producer cells [24]. Second, even WNT3A, a known inducer of ß-catenin-dependent WNT signaling, induced canonical signaling only in about 1.5% of the cells. Thus, we conclude that canonical signaling is activated in a specific subset of HUVECs in response to WNT2, and whether these are more stem cell-like cells or endothelial precursor cells or other mechanisms apply remains to be investigated in future experiments.

However, there was a profound difference in the migratory and invasive capacity of HUVECs in response to WNT2. As more than one-third of HUVECs migrated or invaded within 6–8 h in transwell chambers, this effect cannot be attributed to the sole canonical effect (affecting only 1.5% of the cells) or an eventual secondary effect induced by the canonical signaling. Hence, we conclude that WNT2 might be responsible to induce non-canonical Ca^2+^ and/or planar-cell-polarity pathways in HUVECs. This will be examined in future experiments. The migration promoting effect of WNT2 is well documented in CAFs [24] and smooth muscle cells in atherosclerosis [62], though to the best of our knowledge there is so far no report available on effects of WNT2 on EC migration.

Most importantly, there is a highly significant difference in the angiogenic response of HUVECs to WNT2 expression in stromal fibroblasts. This was determined in a physiologically relevant in vitro assay, which was previously shown to recapitulate many features of in vivo angiogenesis and displayed in vivo-like response to pro- and anti-angiogenic compounds [40]. Moreover, results obtained from primary CAFs and HUVECs derived from different donors showed very high consistency. In addition, the positive effect on angiogenesis when skin fibroblasts devoid of WNT2 expression were forced to express WNT2, in comparison to the reduction of angiogenesis when WNT2 was ablated from colonic CAFs, further support the significance of our findings. Of note, the results from in vitro experiments were recapitulated in a xenograft colon cancer model in mice indicating the in vivo relevance. This was further underscored by the correlation of WNT2 expression with potential angiogenic markers in human colon cancer samples.

The combination of transcriptional analyses and in-depth proteomic secretome profiling of CAFs treated with WNT2-specific or control siRNA provided deeper knowledge about the regulation of secretory proteins by WNT2 in CAFs. In general, more than 1000 proteins were identified in cell culture supernatants of CAFs derived from two different colon cancer patients, including many that have previously been described as stromal biomarkers for CRC. For instance, follistatin-related protein 1 (FSTL1), latent-transforming growth factor beta-binding protein 2 (LTBP2), spondin-2 (SPON2), calumenin (CALU), olfactomedin-like protein 3 (OLFML3), and cadherin-11 (CDH11), described in [63], were highly abundant in our samples. Other reactive stromal markers such as secreted protein acidic and cysteine-rich (SPARC), lysyl oxidase homolog 2 (LOXL2), adipocyte enhancer-binding protein 1 (AEBP1) [64], and insulin-like growth factor-binding protein 7 (IGFBP7) [25, 64] were also present at high abundance in our dataset. We also identified the ECM protein tenascin C (TNC) and the matrix metalloproteinase 3 (MMP3) that were demonstrated to be representative markers for CAFs originating from resident fibroblasts [65]. Most interestingly, of these stromal marker proteins FSTL1, SPON2, LTBP2, AEBP1, and IGFBP7 were significantly downregulated in CAF secretomes when WNT2 was silenced by genetic interference, indicating a role for WNT2 in maintenance of CAF marker expression.

As angiogenesis requires the proteolytic cleavage of ECM to enable invasion of migrating and proliferating ECs, matrix metalloproteinases (MMPs) play a vital role in angiogenesis [66]. MMP regulation by WNT2 in colonic CAFs was already evident on the transcriptional level (Supplementary Table S1 and Supplementary Figure S6) and all MMPs detected by LC-MS (MMP1, -2, -3, -7, -14 and -19) were slightly downregulated in CAFs devoid of WNT2, which was statistically significant in both CAFs for MMP3 and MMP19 (Supplementary Table S2a).

In-depth literature search of the 44 proteins that showed significantly reduced expression in CAF-siWNT2 with a minimum fold change of 1.5 compared to CAF-NTC revealed that 35.6% of the proteins, including connective-tissue growth factor (CTGF), macrophage colony-stimulating factor (M-CSF), and stanniocalcin-2 (STC2), were described to exert a pro-angiogenic effect, while only 15.6% were reported to play an inhibitory role in angiogenesis (see Fig. 6; Table 1). Angiogenesis is a tightly regulated process that is dependent on a multitude of signals and factors, which are held in a constant equilibrium under normal conditions. This balance is disrupted during phases where increased blood supply is needed, e.g., during development, wound healing, or pathological conditions [67]. Therefore, it is not surprising that not only pro-angiogenic factors but also some anti-angiogenic factors are changed by WNT2. However, the regulation of known angiogenic inducers by WNT2 prevails in CAFs of CRC patients, thus leading to a shift in the angiogenic balance towards a pro-angiogenic milieu in the tumor stroma.

Moreover, we also examined the expression of cytokines and growth factors using antibody-based cytokine and bead arrays, as their detection via high-throughput mass spectrometry is limited due to the small protein size and the low abundance of cytokines [56]. The most prominent reduction in expression was observed for IL-6 and G-CSF in CAFs upon depletion of WNT2 in single and in co-cultures with HUVECs. In HUVECs, ectopic expression of WNT2 led to a significant induction of PGF and ANG-2. All these factors have already been reported to display a functional role in blood vessel formation [68–71], whereas the reports on PGF and G-CSF in angiogenesis are conflicting [72, 73]. In our hands, addition of IL-6, G-CSF and PGF in a simple tube formation assay led to enhanced vessel formation and branching in vitro, which further strengthens our hypothesis that WNT2 potently drives pathological angiogenesis in CRC.

## Conclusions

Taken together, our systematic comprehensive analysis of stroma-derived WNT2 function in CRC points to a pivotal role of WNT2 in sustaining of an activated CAF phenotype, which is associated with the maintenance of a pro-angiogenic secretome. We conclude that this in combination with the direct effect of WNT2 on EC migration and invasion contributes to elevated tumor angiogenesis in CRC.

## Electronic Supplementary Material

Below is the link to the electronic supplementary material

Supplementary material 1 (PDF 317 kb)

Supplementary material 2 (PDF 2492 kb)

Supplementary Material 3 (XLSX 77 kb)

Supplementary Material 4 (XLSX 447 kb)

## Data Availability

The mass spectrometry proteomics data have been deposited to the ProteomeXchange consortium (http://proteomecentral.proteomexchange.org) via the PRIDE partner repository and are available with the project accession PXD014019.
