# Supplementary material for: Cancer-associated fibroblast-derived WNT2 increases tumor angiogenesis in colon cancer
Source: Angiogenesis. 2019 Oct 30;23(2):159–77. doi: 10.1007/s10456-019-09688-8 (PMC7160098; doi:10.1007/s10456-019-09688-8)
Supplement: Supplementary file 1 — Supplementary material 1 (PDF 317 kb) [file 10456_2019_9688_MOESM1_ESM.pdf]

**Cancer associated fibroblast-derived WNT2 increases tumor angiogenesis in colon cancer**

Daniela Unterleuthner, Patrick Neuhold, Katharina Schwarz, Lukas Janker, Benjamin Neuditschko, Harini Nivarthi, Ilija Crncec, Nina Kramer, Christine Unger, Markus Hengstschläger, Robert Eferl, Richard Moriggl, Wolfgang Sommergruber, Christopher Gerner and Helmut Dolznig

**corresponding author:** Helmut Dolznig, Institute of Medical Genetics, Medical University of Vienna; helmut.dolznig@meduniwien.ac.at

## Supplementary File S1: Material and Methods

### Secretome profiling by high resolution mass-spectrometry

Three biological replicates of CAF-NTC and CAF-siWNT2 were grown for 72 h after transfection with the respective siRNAs. They were washed twice with 1 x Dulbecco´s Phosphate Buffered Saline (DPBS, #17-512F, Lonza) and were cultivated for 6 h under serum-free conditions in RPMI 1640 (Gibco™, Life technologies). Supernatants were collected, filtered through 0.2 µm sterile-filters (#514-1111P, Whatman™, GE Healthcare, Chicago, IL, USA) and precipitated in ethanol at a dilution factor of 1:5 overnight at -20°C. After centrifugation at 4536 g precipitated proteins were dissolved in sample buffer containing 7.5 M Urea, 1.5 M Thiourea, 4% 3-[(3-Cholamidopropyl)dimethylammonio]-1-propanesulfonate (CHAPS), 0.05% sodium dodecyl sulfate (SDS) and 100 mM Dithriothreitol (DTT). Protein concentrations were assessed via a Bradford Protein Assay (Bio-Rad Laboratories, Hercules, CA, USA). A filter-based in-solution digest using 10 kDa molecular weight cut-off centrifugal filter units (#MRCPRT010, Merck Millipore, Burlington, MA, USA) and a Trypsin/Lys C Mix (Promega) was performed with 20 µg of each sample as described previously[1]. In brief, proteins were concentrated on pre-wetted 10 kDa molecular weight cut-off centrifugal filter units (#MRCPRT010, Merck Millipore, Burlington, MA, USA). Proteins were reduced with DTT, alkylated with iodoacetamide (IAA) and washed with 50 mM ammonium bicarbonate buffer. After the tryptic digest, which was performed at 37°C overnight using a Trypsin/Lys-C-Mix (#V5071, Promega), peptides were dried via vacuum centrifugation for approximately 1.5 h and were stored at -20°C until they were analyzed via LC-MS. Prior to analysis peptides were solubilized in 5 µl of 30% formic acid (FA) containing 10 fmol of four synthetic standard peptides each and further diluted with 40 µl of mobile phase A, consisting of 97,9% MS grade H2O, 2% acetonitrile (ACN) and 0.1% FA. In technical duplicates, 1 µl per sample were injected into the nano-HPLC system (Dionex Ultimate 3000, Thermo Fisher Scientific, Waltham, MA, USA) at a flow rate of 10 µl/min using mobile phase A. Peptides were loaded onto a 2 cm x 100 µm C18 precolumn and eluted onto a 50 cm x 75 µm Prepmap100 analytical column. The 85 min methods applied a 43 min gradient from 7% to 40% mobile phase B (79,9% ACN, 20% H2O, 0.1% FA)

at a flow rate of 300 nl/min. MS scans on the Q Exactive Orbitrap, coupled to the nanoLC system with a nanospray ionsource, were performed with a resolution of 70,000 at m/z 200 in a range from m/z 400 to 1,400. MS/MS scans of the peptide ions selected using a top 8 method were analyzed at a resolution of 17,500 at m/z 200. HCD fragmentation was applied at 30% normalized collision energy. For protein identification and label-free quantification (LFQ) the MaxQuant software (v 1.6.0.2) utilizing the Andromeda search engine and the Perseus statistical analysis package (v.1.6.0.2) were employed.2,3 Proteins were identified by searching against the UniProt database for human proteins (version 03/2018, restricted to reviewed entries only) with 20,316 entries, applying a peptide mass tolerance of 50 ppm for the first and 25 ppm for the main search (??). A minimum of two peptide identifications, at least one of them being a unique peptide, was required for valid protein identification. Protein isoforms were summarized into protein groups by the software. The false discovery rate (FDR) was set to 0.01 on both protein and peptide level and was determined by the target-decoy approach. For the search carbamidomethylation cysteines was defined as a fixed modification, methionine oxidation and C-terminal protein acetylation as variable modifications. For each peptide, considering trypsin/P for protease specificity, a maximum of two missed cleavages and two modifications was allowed. An alignment window of 10 min with a match time window of 1 min was set for the function “Match between runs”. Statistical analysis of the data obtained by the MaxQuant search was performed with the Perseus software. Reverse sequences, potential contaminants as well as proteins only identified by site were excluded. After LFQ values were logarithmized to base 2, they were filtered for valid values that were identified in at least 70% of the measurements in at least one group (CAF-NTC or CAF-siWNT2). For enabling statistical analysis, missing values were replaced from the data´s Gaussian distribution of the respective sample with a down shift of 1.8 and a width of 0.3. Changes in protein abundance between the different samples were determined by comparison of the LFQ values of each protein using two-sided t-tests, considering proteins to be significantly regulated with a p-value of < 0.05. Additionally, a global permutation-based FDR correction was applied for multi-parameter testing. Processing of the same protein amount of 20 µg per sample served as normalization. A principal component analysis (PCA) was performed with the Perseus software. For enrichment analysis based on gene ontology (GO) terms for biological processes the DAVID functional annotation tool, version 6.8, was used.4,5

**RT2 profiler Arrays**

RNA was isolated as described for RT-qPCR; cDNA synthesis of 500 ng RNA was performed using the RT² First Strand Kit (Qiagen, #330401) according to the manufacturer´s protocol with genomic DNA elimination. cDNA was diluted by addition of 91 µl RNAse free water per sample and 2 x RT² SYBR Green Mastermix (Qiagen, #330503) was used for the real-time PCRs on an Applied

Biosystems™ StepOnePlus™ Real-Time PCR System (Applied Biosystems, Waltham, MA, USA). The following RT2 Profiler PCR Arrays were used: Human Angiogenic Growth Factors (#PAHS-072Z), Human Extracellular Matrix & Adhesion Molecules (#PAHS-013Z), Human WNT Signaling Pathway (#PAHS-043Z), Human WNT Signaling Targets (#PAHS-243Z). Data were analyzed using the web-based GeneGlobe Data Analysis Center provided on the QIAGEN homepage. The sum of all signals (including the housekeeping genes) from individual plates were used for plate to plate normalization, the results were ranked by the mean signaling strength of one condition and heatmaps were generated using Excel conditional formatting. Measurements were performed at least in three biological replicates and differences in signaling activity were tested for statistical significance.


1 Bileck, A., Kreutz, D., Muqaku, B., Slany, A. & Gerner, C. Comprehensive assessment of proteins regulated by dexamethasone reveals novel effects in primary human peripheral blood mononuclear cells. *Journal of proteome research* **13**, 5989-6000, doi:10.1021/pr5008625 (2014).

2 Cox, J. & Mann, M. MaxQuant enables high peptide identification rates, individualized p.p.b.-range mass accuracies and proteome-wide protein quantification. *Nature biotechnology* **26**, 1367-1372, doi:10.1038/nbt.1511 (2008).

3 Cox, J. & Mann, M. 1D and 2D annotation enrichment: a statistical method integrating quantitative proteomics with complementary high-throughput data. *BMC bioinformatics* **13 Suppl 16**, S12, doi:10.1186/1471-2105-13-S16-S12 (2012).

4 Huang da, W., Sherman, B. T. & Lempicki, R. A. Systematic and integrative analysis of large gene lists using DAVID bioinformatics resources. *Nature protocols* **4**, 44-57, doi:10.1038/nprot.2008.211 (2009).

5 Huang da, W., Sherman, B. T. & Lempicki, R. A. Bioinformatics enrichment tools: paths toward the comprehensive functional analysis of large gene lists. *Nucleic acids research* **37**, 1-13, doi:10.1093/nar/gkn923 (2009).
