# Supplementary material for: Cancer-associated fibroblast-derived WNT2 increases tumor angiogenesis in colon cancer
Source: Angiogenesis. 2019 Oct 30;23(2):159–77. doi: 10.1007/s10456-019-09688-8 (PMC7160098; doi:10.1007/s10456-019-09688-8)
Supplement: Supplementary file 2 — Supplementary material 2 (PDF 2492 kb) [file 10456_2019_9688_MOESM2_ESM.pdf]

Cancer associated fibroblast-derived WNT2 increases tumor angiogenesis in colon cancer

Daniela Unterleuthner, Patrick Neuhold, Katharina Schwarz, Lukas Janker, Benjamin Neuditschko, Harini Nivarthi, Ilija Crncec, Nina Kramer, Christine Unger, Markus Hengstschläger, Robert Eferl, Richard Moriggl, Wolfgang Sommergruber, Christopher Gerner and Helmut Dolznig

corresponding author: Helmut Dolznig, helmut.dolznig@meduniwien.ac.at, Tel. +43 1 40160 56502

Supplementary Figures S1 – S8

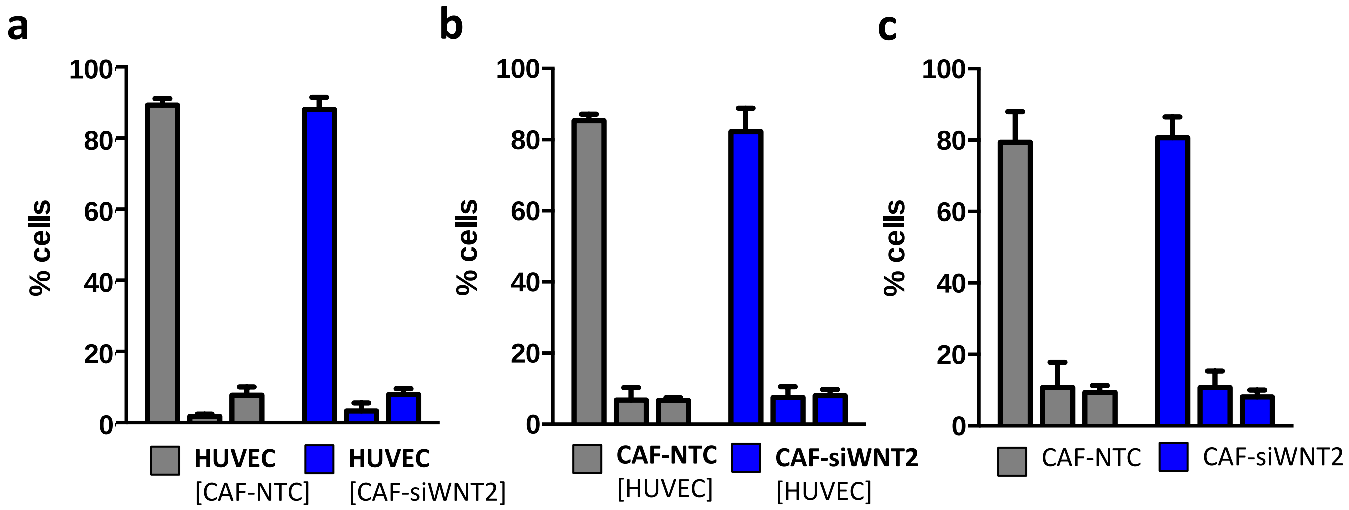

Supplementary Figure S1: ***Wnt2 does not change cell cycle progression in HUVECs and CAFs.*** Cell cycle profiles of CAF#2 and HUVEC were obtained by flow cytometry analysis of monolayer cells by EdU incorporation (20 min pulse) and 7AAD staining. Percentages of G1, S, G2/M phase were determined. Bars are mean ±SEM; data are from three biological replicates. HUVECs in co-culture with CAF either depleted of WNT2 by siRNA-mediated knockdown (CAF-siWNT2) or transfected with non-targeting control (CAF-NTC). The EC marker CD31 was used to distinguish the two cell types in the co-cultures. Cell cycle distribution of HUVEC cells (gated as $CD31^+$) is shown in **a**, whereas the profiles of the CAFs ($CD31^-$) are shown in **b**. Co-cultures are indicated by listing both cell types, bold letters indicate cells analyzed; square brackets indicate the cells not being analyzed. **c** Monoculture of CAFs grown in monolayers.

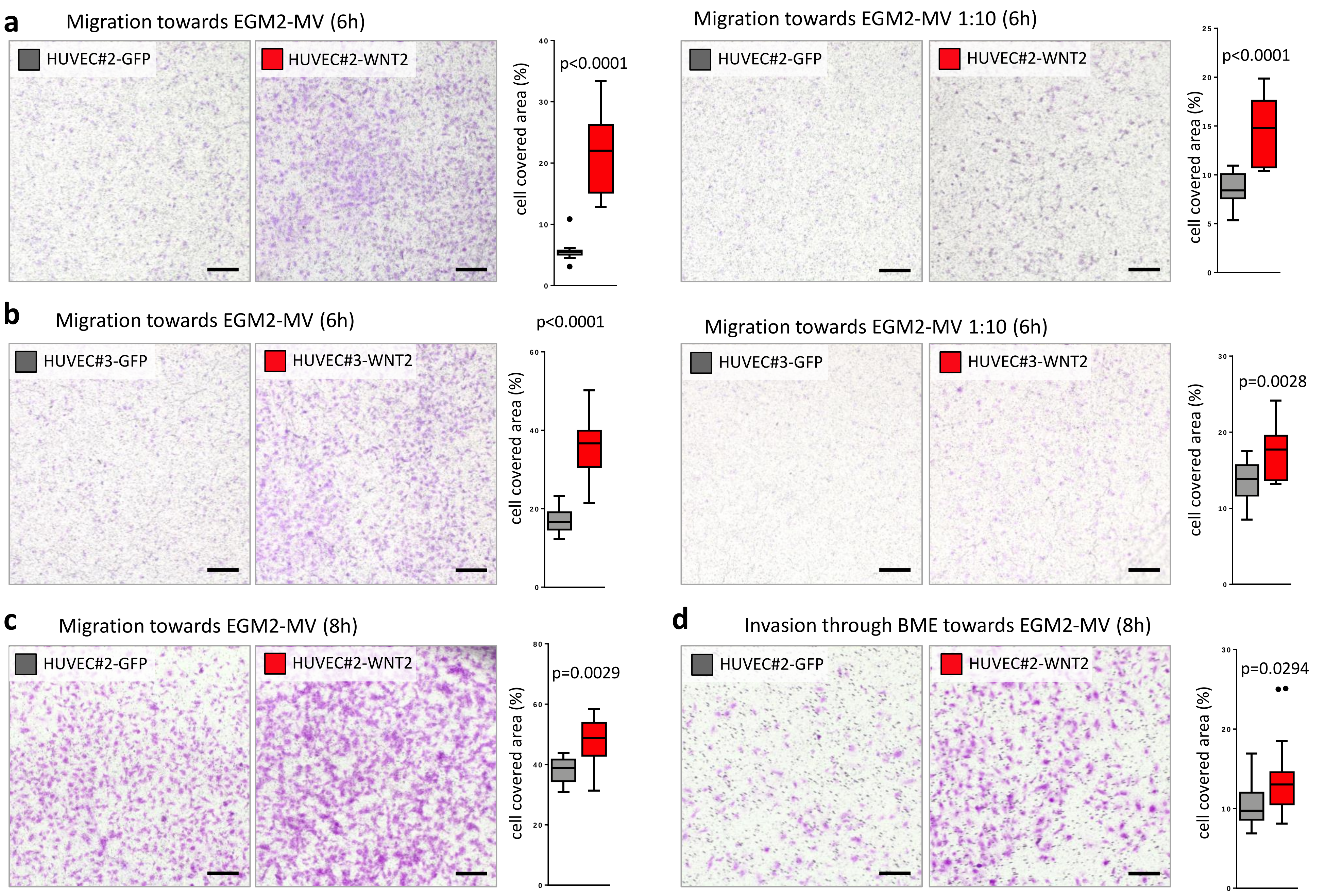

Unterleuthner et al. Supplementary Figure S2

Supplementary Figure S2: WNT2 *significantly induces migration and invasion of HUVEC.* Migration and invasion of HUVEC-WNT2 (red) from factor-free basal medium towards full EGM™-2MV or DMEM plus 10% EGM™-2MV in comparison to HUVEC-GFP (gray) was assessed using transwell inserts with 5.0 µm or 8.0 µm pore sizes. Representative pictures of crystal violet stained migrated cells (**a,b,c**) or invaded through BME-coated inserts (**d**) at the lower surface of the transwell membrane are shown on the left, membrane coverage as quantification of migration/invasion of HUVECs is shown in Whisker-box plots on the right. Horizontal lines in the plots indicate the median, boxes represent the interquartile range (IQR) between the 25th and 75th percentile, whiskers extend to 1.5 times the IQR. Outliers are displayed by dots; p values are indicated. For all migration experiments except for the one shown in C transwell inserts with 5.0 µm pore size were used and all experiments were carried out at least in duplicates. For the experiment shown in c migration inserts with 8.0 µm pore size were used as a control for invasion experiments as invasion inserts have 8.0 µm pore size.

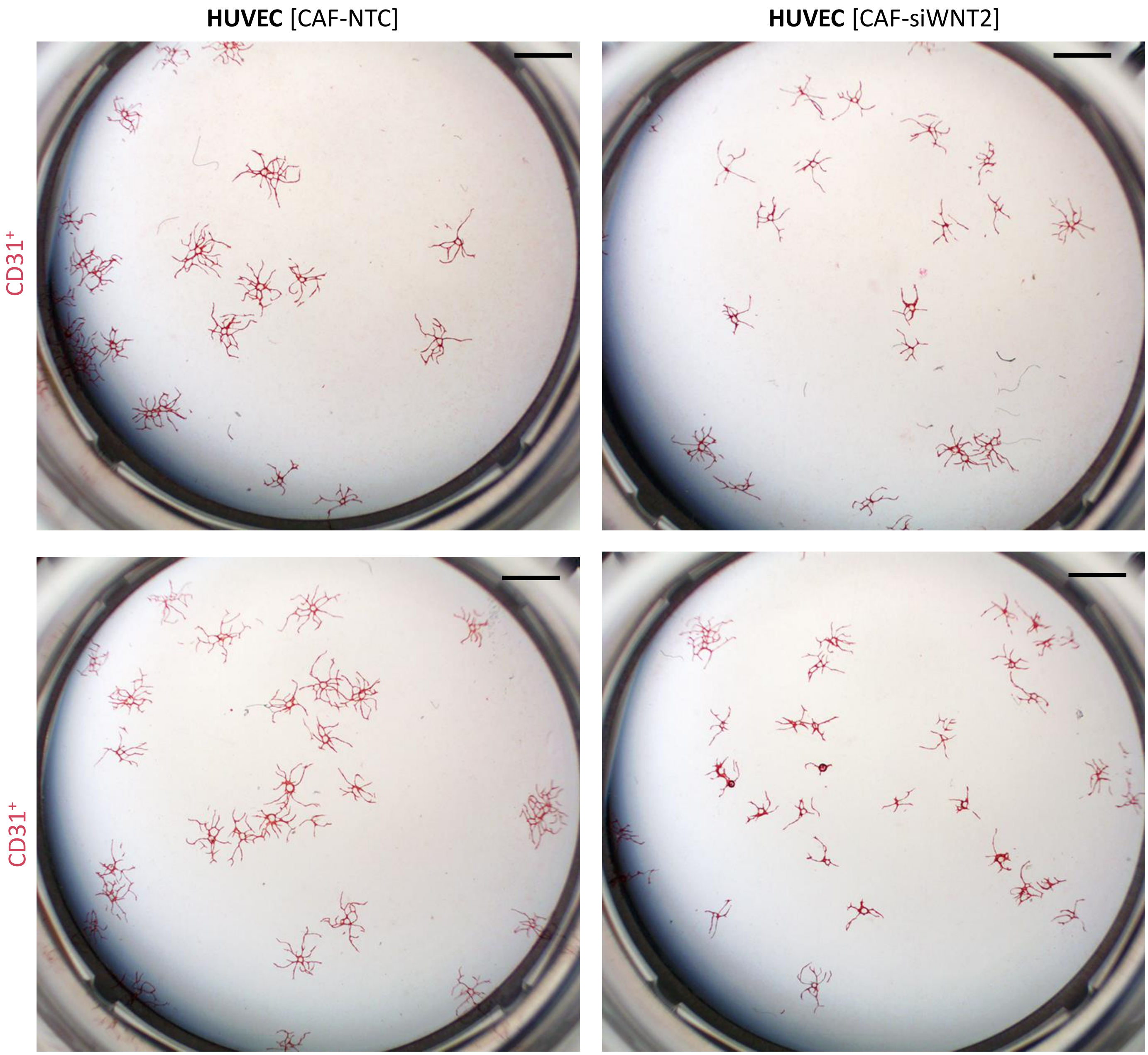

Supplementary Figure S3: *CD31+ endothelial structures*. Representative overview pictures of EC structures formed in 24-wells in co-cultures with CAF-NTC or CAF-siWNT2 (corresponding to the results presented in Figure 3). CAF#2 were used for this experiment. HUVECs were immunohistochemically stained with the endothelial marker CD31 (red) to distinguish them from densely seeded CAFs. Scale bar represents 3 mm.

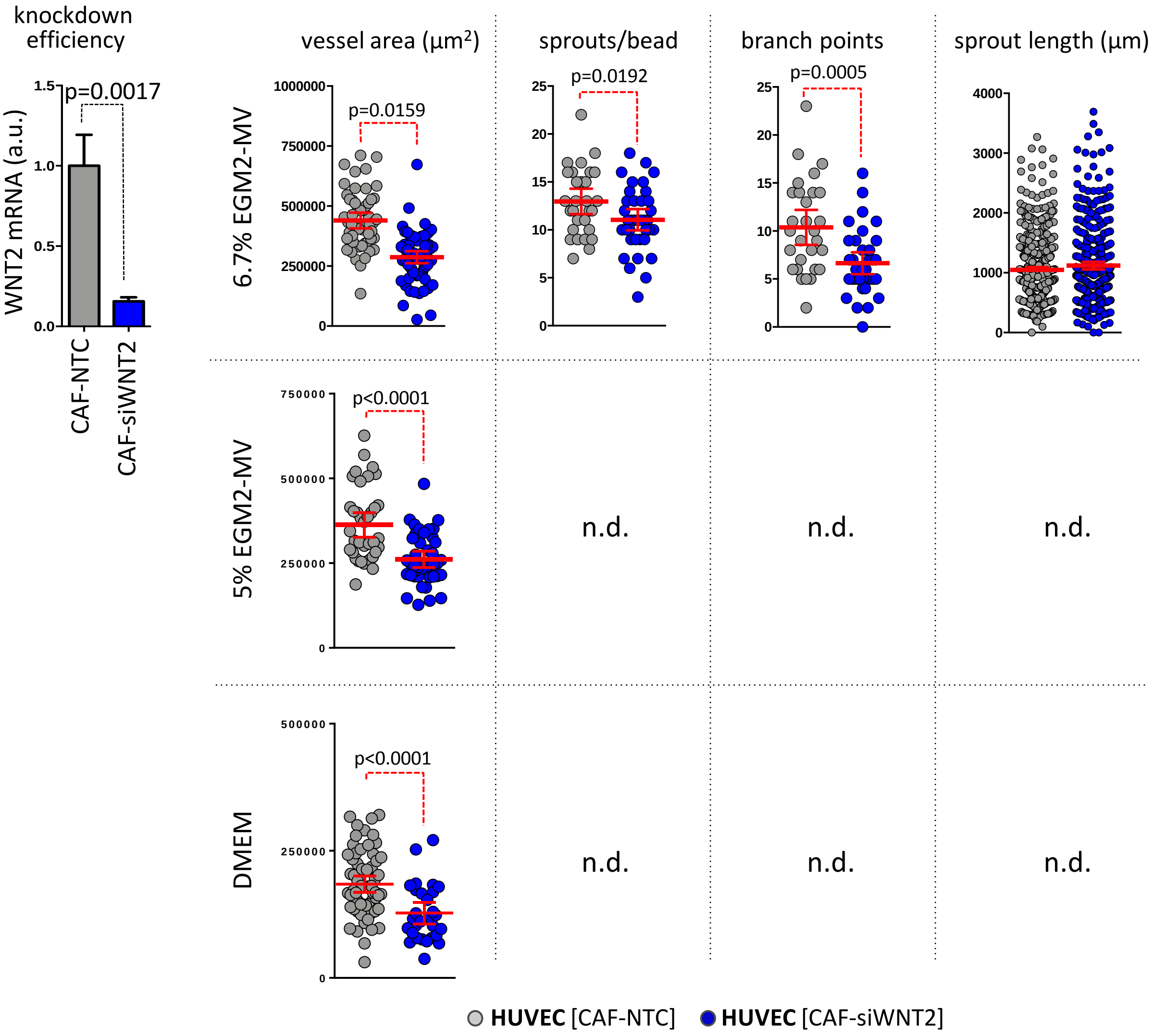

Supplementary Figure S4a***: Fibroblast-derived WNT2 induces vessel growth and sprouting in a 3D angiogenesis co-culture assay*****.** The data shown in this figure were obtained by using the same CAF#1 and HUVECs that were used for the data presented in Figure 3. CAFs endogenously expressing WNT2 (CAF-NTC, gray) or with a WNT2 knockdown (CAF-siWNT2, blue) were co-cultivated with HUVEC-coated microcarrier beads in additional medium compositions, DMEM supplemented either with 6.7%, 5% or 0% of EGM2-MV. WNT2 depletion was evaluated by RT-qPCR. As the same cells were used as in Figure 3 the graphical representation of WNT2-mRNA is shown here again for clarity. Image processing was used to quantify vessel areas, sprout numbers, branch points, and sprout length per bead {Unterleuthner, 2017 #1}. Red horizontal lines indicate the mean; error bars are SEM; 6.7% EGM2-MV: CAF-NTC, n=54; CAF-siWNT2, n=68; 5% EGM2-MV: CAF-NTC, n=63; CAF-siWNT2, n=29; DMEM: CAF-NTC, n=37; CAF-siWNT2, n=42; P values are given.

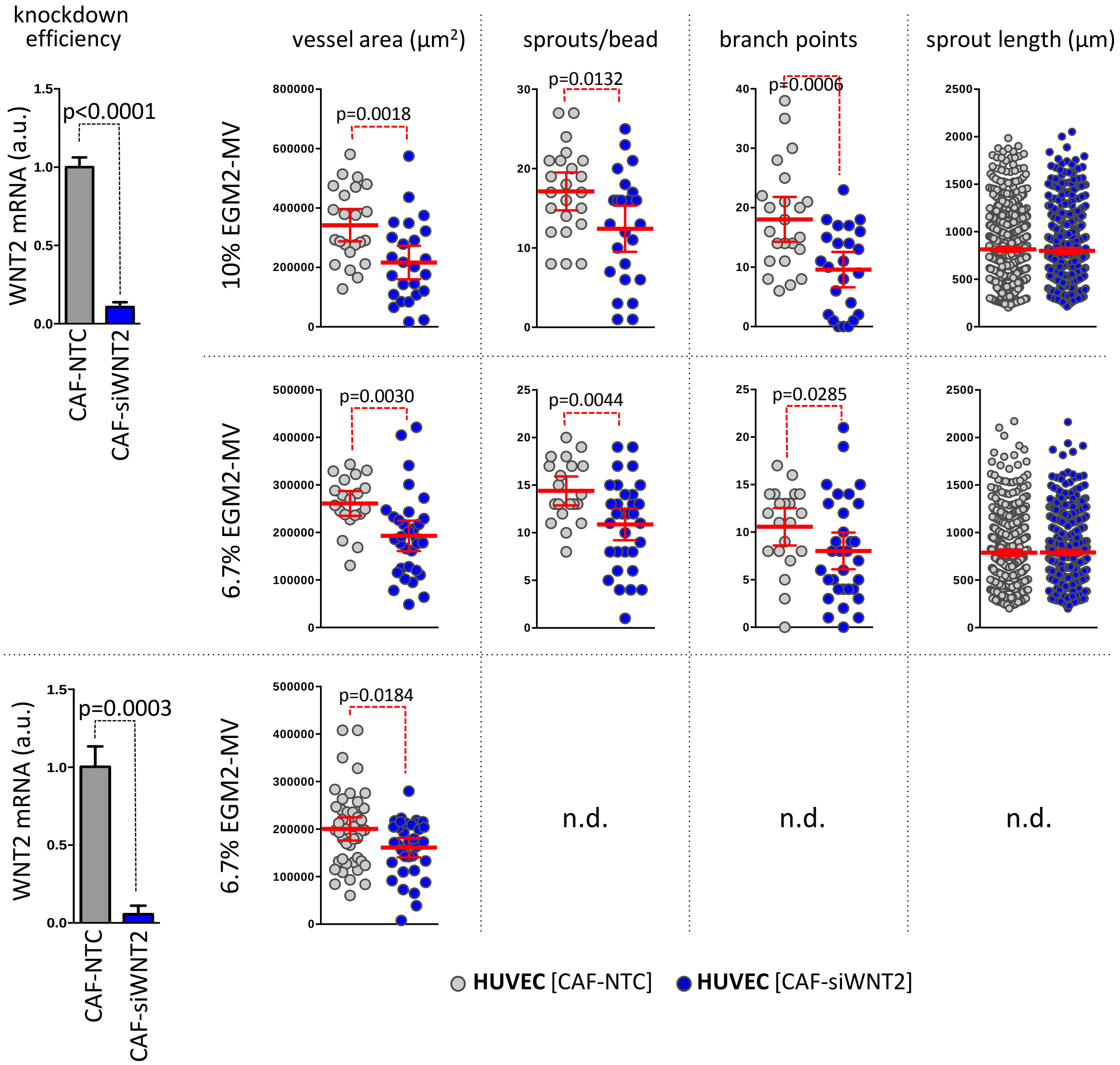

Supplementary Figure S4b*: Fibroblast-derived WNT2 induces vessel growth and sprouting in a 3D angiogenesis co-culture assay*. The data shown in this figure were obtained by using CAF#2 and HUVECs. CAFs endogenously expressing WNT2 (CAF-NTC, gray) or with a WNT2 knockdown (CAF-siWNT2, blue) were co-cultivated with HUVEC-coated microcarrier beads in DMEM supplemented with either 10% or 6.7% of EGM2-MV. WNT2 depletion was evaluated by RT-qPCR, graphical representation is shown in bar graphs. Quantifications of vessel areas, sprout numbers, branch points, and sprout length per bead are depicted in scatter dot plots. Red horizontal lines indicate the mean; error bars are SEM; first experiment: 10% EGM2-MV: CAF-NTC, n=23; CAF-siWNT2, n=25; 6.7% EGM2-MV: CAF-NTC, n=20; CAF-siWNT2, n=32; second experiment: CAF-NTC, n=44; CAF-siWNT2, n=35; P values are given.

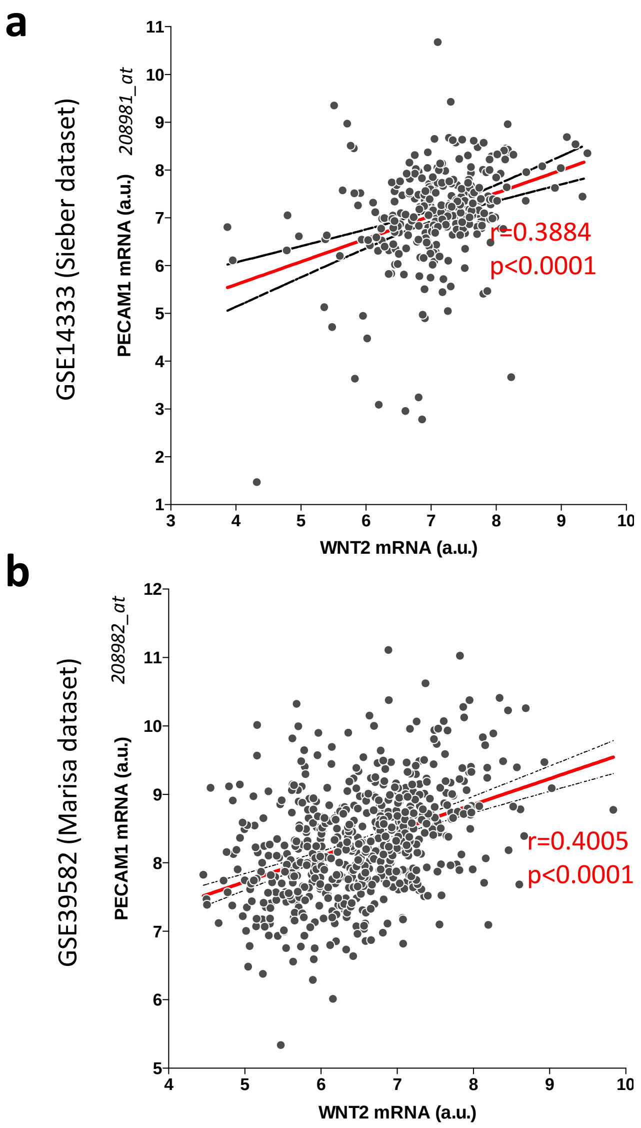

Supplementary Figure S5: ***WNT2 and PECAM (CD31) expression correlate in datasets of human CRC.*** Correlation of WNT2 and PECAM1 (CD31) mRNA expression in 290 and 585 CRC patients using the (**a**) Sieber (GSE14333) or (**b**) Marisa (GSE39582) datasets, respectively. Gray dots represent individual samples; red line illustrates linear regression.

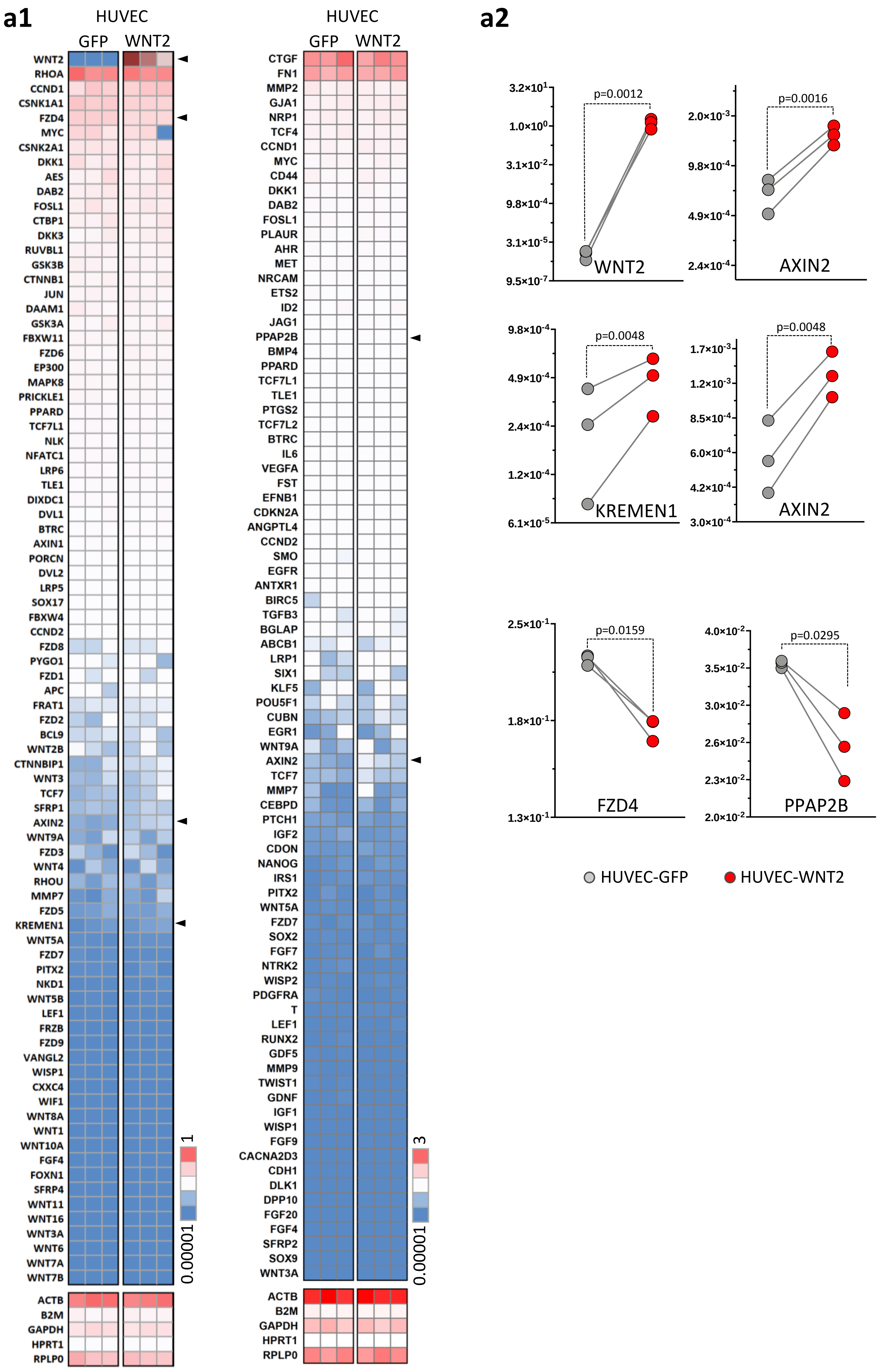

Unterleuthner et al. Supplementary Figure S6a

Supplementary Figure S6a: ***Gene expression profiling of components of WNT signaling pathways and targets in HUVECs*****.** mRNA expression levels of 84 genes specific for WNT signaling pathways and targets were assessed by quantitative RT-PCR in HUVEC-WNT2 in comparison to GFP-expressing controls in three biological replicates. Expression of the genes was normalized to the sum of expression of all genes including the housekeeping genes. **a1** Heatmaps of WNT pathway (left) and WNT target (right) gene expression. The genes of the biological replicates were ranked based on the mean expression in all six conditions. Blue indicates low expression, red indicates high expression, white represents intermediate expression; see color scales. Arrowheads: significant changes. **a2** Dot plots of the significantly regulated genes with FC>1.4 and <0.7. P-values are indicated.

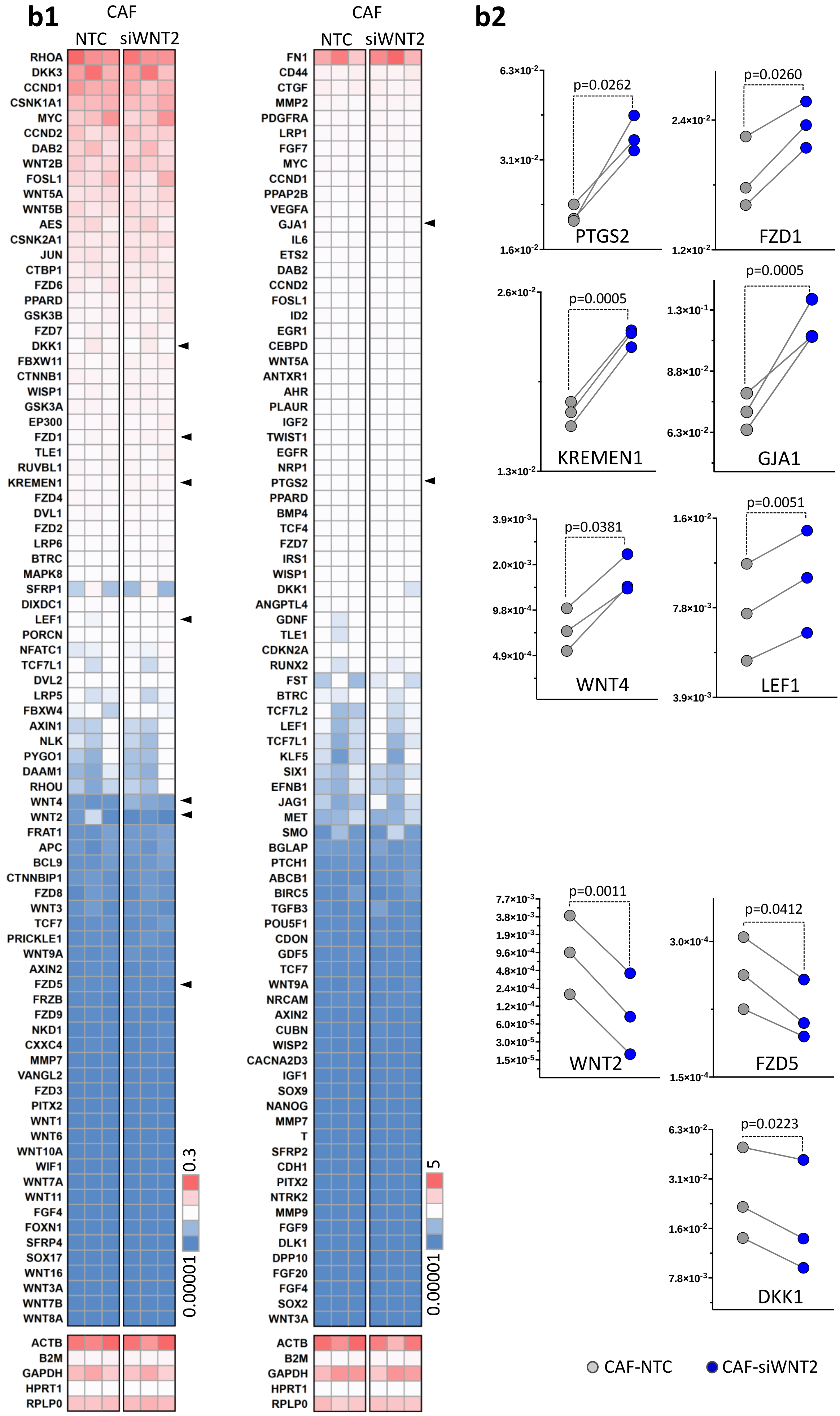

Unterleuthner et al. Supplementary Figure S6b

Supplementary Figure S6b: ***Gene expression profiling of components of WNT signaling pathways and targets in CAFs.*** mRNA expression levels of 84 genes specific for WNT signaling pathways and targets were assessed by quantitative RT-PCR in CAF-siWNT2 in comparison to CAF-NTC controls in three biological replicates. Expression of the genes was normalized to the sum of expression of all genes including the housekeeping genes. **b1** Heatmaps of WNT pathway (left) and WNT target (right) gene expression. The genes of the biological replicates were ranked based on the mean expression in all six conditions. Blue indicates low expression, red indicates high expression, white represents intermediate expression; see color scales. Arrowheads: significant changes. **b2** Dot plots of the significantly regulated genes with FC>1.4 and <0.7. P-values are indicated.

c1

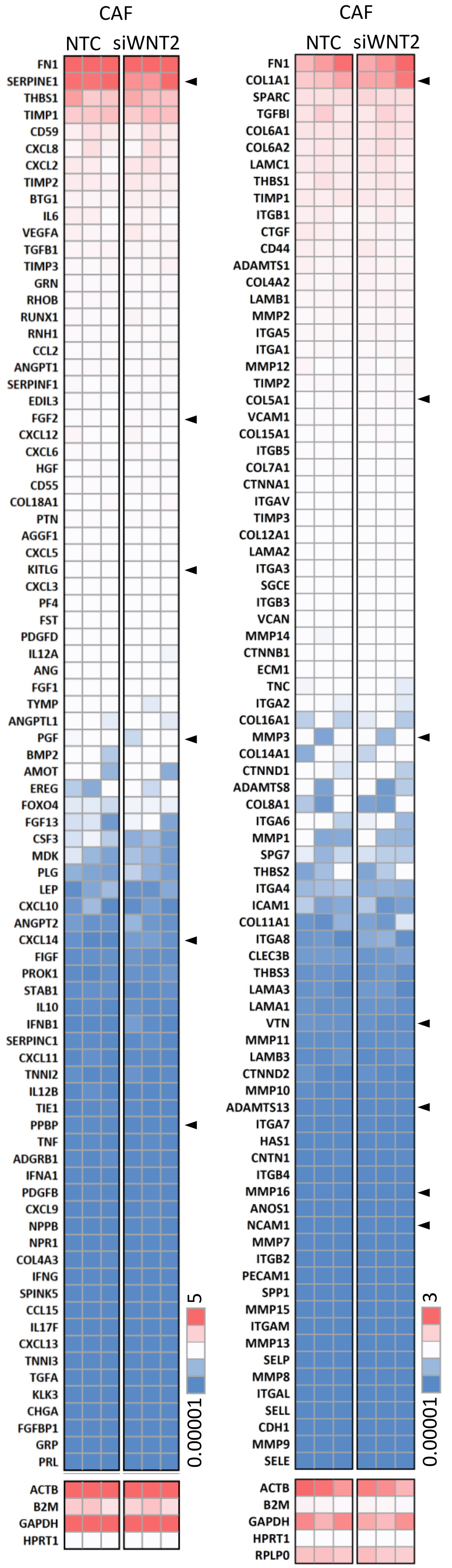

c2

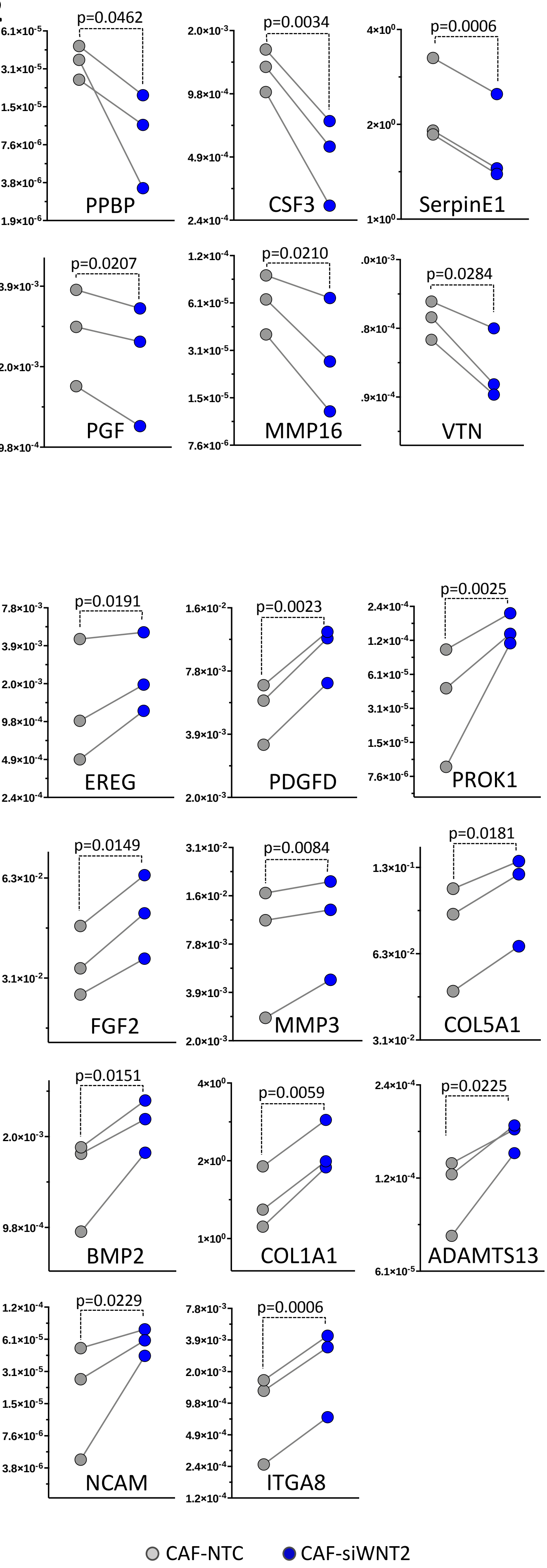

Unterleuthner et al. Supplementary Figure S6c

Supplementary Figure S6c: ***Gene expression profiling of angiogenic growth factors and ECM and cell adhesion-related genes in CAFs*****.** mRNA expression levels of 84 genes specific for angiogenic growth factors and ECM and adhesion molecules were assessed by quantitative RT-PCR in CAF-siWNT2 in comparison to CAF-NTC controls in three biological replicates. Expression of the genes was normalized to the sum of expression of all genes including the housekeeping genes. **c1** Heatmaps of angiogenic growth factor (left) and and ECM and adhesion molecule (right) gene expression. The genes of the biological replicates were ranked based on the mean expression in all six conditions. Blue indicates low expression, red indicates high expression, white represents intermediate expression; see color scales. Arrowheads: significant changes. **c2** Dot plots of the significantly regulated genes with FC>1.4 and <0.7. P-values are indicated

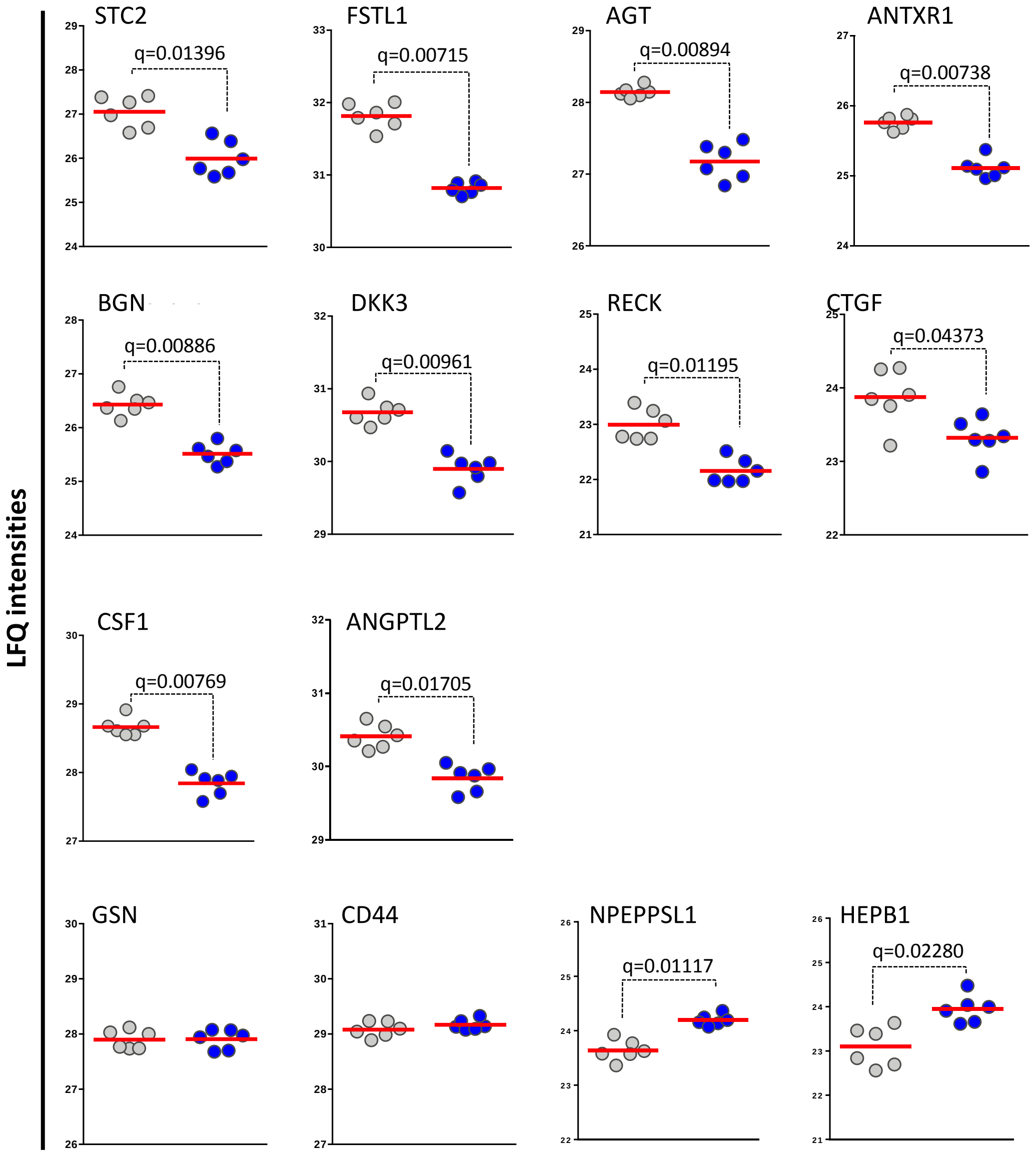

Supplementary Figure S7: ***Secretome profiling of CAFs in presence (CAF-NTC) or absence of WNT2 (CAF-siWNT2).*** Scatter dot plots of LFQ intensities of examples for significantly down- or upregulated and not-regulated proteins in upon knockdown of WNT2 (CAF-siWNT2) compared to NTC-siRNA treated controls (CAF-NTC) as determined by LC-MS-based secretome profiling. Data from all biological and technical replicates of CAF#1 are depicted. Red lines represent the mean. Q-values of multi-parameter corrected significance tests are indicated.

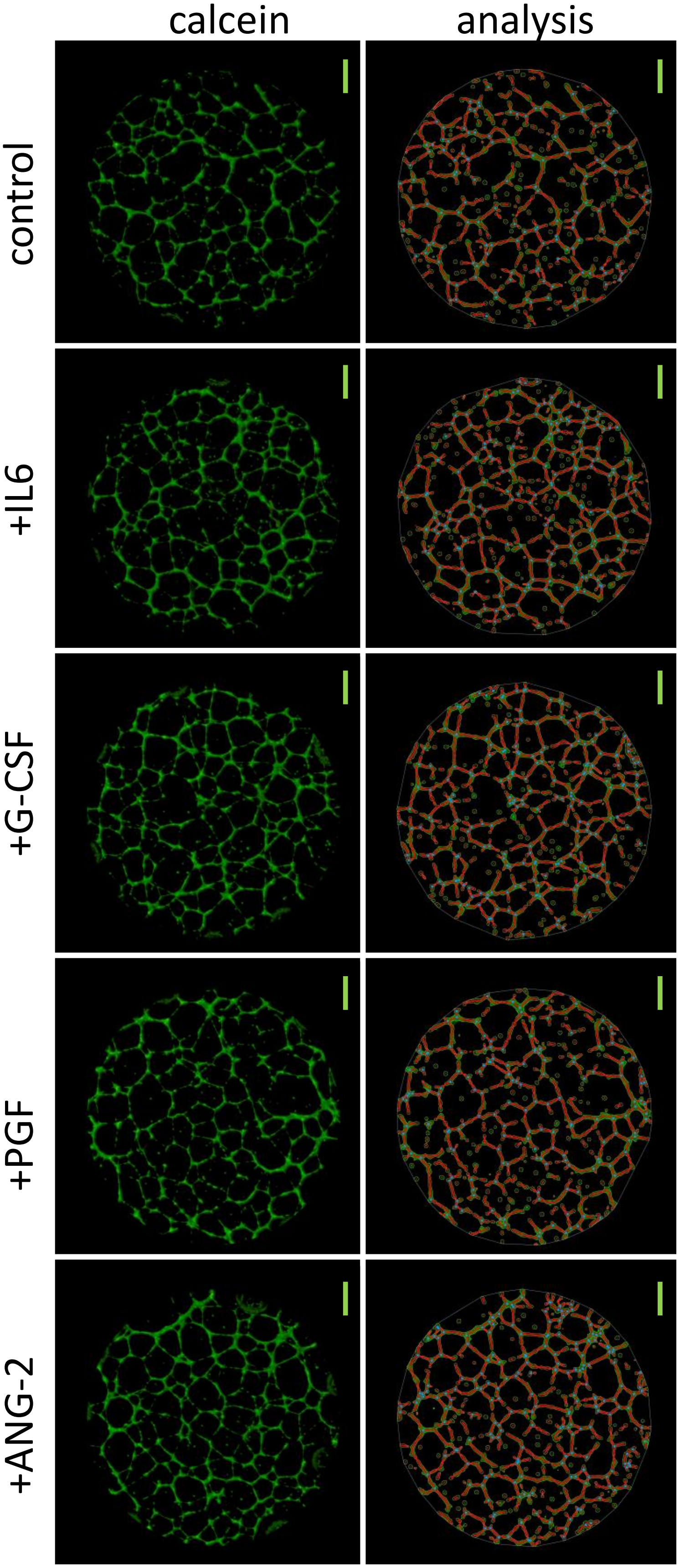

Supplementary Figure S8: ***Matrigel tube formation assay.*** Representative images of the tubes formed on matrigel in DMEM supplemented with 5%FCS and 10% of EGM™-2 MV with either 20 ng/mL of IL6, G-CSF or PGF or 100 ng/ml of ANG-2 added. The left panel shows HUVECs on matrigel stained with calcein, the corresponding result image created via automated analysis of the structures by the open source software AngioTool is shown on the right. The outlines of the tubes are shown in yellow, the red lines mark the skeletonized tubes and branching points are indicated in blue. Scale bars represent 500 µm.
